# Personality and brain contribute to academic achievements of medical students

**DOI:** 10.3389/fnins.2022.964904

**Published:** 2022-09-06

**Authors:** Yingying Xie, Congcong Yuan, Mengru Sun, Jie Sun, Ningnannan Zhang, Wen Qin, Feng Liu, Hui Xue, Hao Ding, Sijia Wang, Jinyan He, Lizhi Hu, Xiaoxia Li, Chunshui Yu

**Affiliations:** ^1^Department of Radiology and Tianjin Key Laboratory of Functional Imaging, Tianjin Medical University General Hospital, Tianjin, China; ^2^Tianjin Medical University, Tianjin, China

**Keywords:** brain function, brain structural, medical education, academic achievements, personality, mediation analysis

## Abstract

There are many factors that influence the academic achievements of medical students, but how personality and brain modulate the academic achievements of medical students remains unclear. The study collected the personality, brain imaging, and academic data from 448 medical students at Tianjin Medical University with admission time between 2008 and 2017. Four types of academic achievements, including behavioral and social sciences, clinical sciences and skills, basic biomedical sciences, and scientific methods, were assessed by the academic records of 58 courses. Personality was evaluated by Tridimensional Personality Questionnaire and Neuroticism Extraversion Openness Personality Inventory. Brain structural and functional properties, including gray matter volume, spontaneous brain activity and functional connectivity, were computed based on magnetic resonance imaging (MRI). Linear regression was used to evaluate the associations between personality and academic achievements. A voxel-wise correlation was used to identify areas of the brain where structural and functional properties were associated with academic achievements. Mediation analysis was used to test whether brain properties and personality independently contribute to academic achievements. Our results showed that novelty seeking (NS) was negatively correlated, and conscientiousness was positively correlated with all types of academic achievements. Brain functional properties showed negatively correlated with academic achievement in basic biomedical sciences. However, we did not find any mediation effect of the brain functional properties on the association between personality (NS and conscientiousness) and academic achievement in basic biomedical sciences, nor mediation effect of the personality (NS and conscientiousness) on the association between brain functional properties and academic achievement in basic biomedical sciences. These findings suggest that specific personality (NS and conscientiousness) and brain functional properties independently contribute to academic achievements in basic biomedical sciences, and that modulation of these properties may benefit academic achievements among medical students.

## Introduction

Academic achievements of medical students represent the mastery of knowledge of medical science and related disciplines and are of great significance to the professional development of medical students. A variety of factors may influence the academic achievements of college students, such as age, sex, socioeconomic status, physical and mental health, intelligence, personality, interests, attitudes, behaviors and motivations, extracurricular activities, high school grades, study habits and methods, study time, teaching methods, teaching, and learning environments (De Luca et al., 2016; Partido and Stafford, 2018; Ahmady et al., 2019; Dzubur et al., 2020; Kusnierz et al., 2020; Al-Momani, 2021). However, only a few of these factors, such as study skills, attitudes, behaviors and motivations, time management, physical activity, personality, and coping strategies, were investigated and associated with the academic achievements of medical students (Ahmady et al., 2019; Hou et al., 2020; Lateef Junaid et al., 2020; Al-Momani, 2021).

Personality refers to individual differences in characteristic patterns of thinking, feeling, and behavior. The PPKI theory, which postulates intelligence as process, personality, knowledge and interest (Ackerman, 1996), proposes that personality plays a critical role in knowledge development, guiding a student’s choice and adherence to learning. Indeed, many studies have reported associations between personality traits and academic achievements, some of which have found associations between personality traits and overall or specific academic achievements (Hoschl and Kozeny, 1997; Vedel, 2014; Marcela, 2015; Bhagat et al., 2016; Mccredie and Kurtz, 2019; Dzubur et al., 2020; Kusnierz et al., 2020). Medical students must study different types of courses, which may be related to different personality traits. However, among medical students, the associations between personality traits and different types of academic achievements remain unknown.

Many factors that influence on academic achievements of medical students have been associated with human brain structural and functional properties. For example, understanding patterns of brain dominance could help improve how medical students teach and learn (Suresh et al., 2020); functional connectivity is associated with academic achievement in reading and is regulated through cognitive control abilities (Jolles et al., 2020); working memory is related to activity in frontal and parietal regions (Eriksson et al., 2015); and long-term memory is associated with the hippocampus (Sawangjit et al., 2018); the motivation to learn is related to the activity in the putamen (Gao et al., 2021). However, few studies have investigated the relationship between structural and functional properties of the human brain and the academic achievements of medical students. Besides, neural substrates are correlated with underlying personality traits and brain properties may help to predict one’s personality (Cai et al., 2020; Kabbara et al., 2020). Since both personality and brain may influence the academic achievements in medical students, and brain properties are associated with personality, it remains unclear how personality and brain structural and functional properties affect different types of academic achievements, and whether there are mediation effects among brain properties, personality, and academic achievements.

In this study, we aimed to answer three questions: (a) whether the academic achievements in different types of courses are correlated with different personality traits; (b) the structural and/or functional properties of which brain regions are correlated with different types of academic achievements; and (c) whether brain properties and personality independently contribute to academic achievements.

**FIGURE 1:**
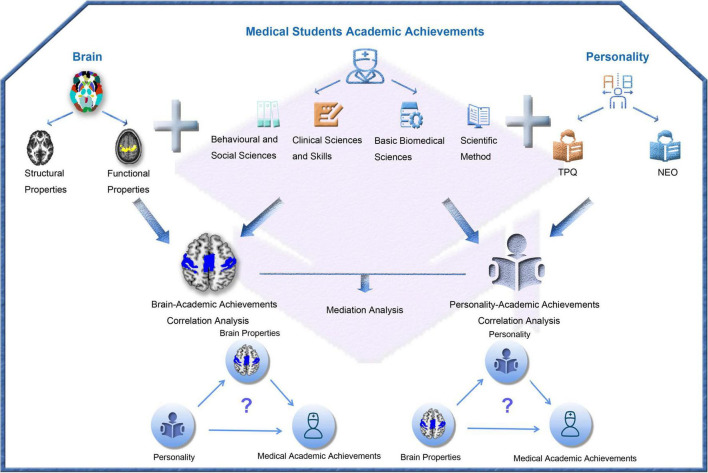
Flowchart of the study design. TPQ, tridimensional personality questionnaire; NEO, neuroticism Extraversion openness personality inventory.

To answer these questions, we categorized 58 courses into four types (behavioral and social sciences, clinical sciences and skills, basic biomedical sciences, and scientific method) and calculated the grade point average (GPA) for each course as its academic achievement. The personality traits of each student were assessed using two commonly used questionnaires: the tridimensional personality questionnaire (TPQ) and Neuroticism Extraversion Openness (NEO) Personality Inventory (Cloninger et al., 1991; Costa and Mccrae, 1992; Caprara et al., 1993). The brain structural and functional properties were evaluated by grey matter volume (GMV), regional homogeneity (ReHo), and amplitude of low frequency fluctuation (ALFF) of spontaneous brain activity, and functional connectivity density and strength (FCD and FCS) that were derived from multi-modality MRI data. In 448 medical students with personality, academic achievements and brain MRI data, we investigated correlations of different personality traits and brain imaging measures with different types of academic achievements. For personality traits and brain imaging measures that were correlated with academic achievements, we performed mediation analysis to clarify whether there were mediation effects among brain properties, personality and academic achievements. The system flow of the study design is shown in Figure 1.

## Materials and methods

### Participants

A total of 448 medical students (153 males, 295 females; age: 22.2 ± 1.8 years, range: 18–29°years; education: 15.6 ± 1.6°years) were recruited from Tianjin Medical University with admission time from 2008 to 2017, with different degrees (Master, *N* = 71; Bachelor, *N* = 375; Doctor, *N* = 2), and the major of all students were Clinical Medicine according to the Undergraduate Specialty Catalog of Higher Institutions (the 2020 revision) which was released by the National Educational Commission of China. Detailed inclusion and exclusion criteria can be found in Supplementary Table 1. This study was approved by the ethics committee of Tianjin Medical University General Hospital and all participants signed written informed consent before the experiment. The assessment time for each subject is detailed in Supplementary Figure 1 and Supplementary Table 2.

### Academic achievements

Firstly, 58 courses were included based on the following criteria: (a) only compulsory courses and restrictive courses are included; (b) students complete courses at Tianjin Medical University and obtain valid scores; and (c) the number of students enrolled in each course is no less than 5% of the total number of students, taking into account the bias caused by the number of students in each course and reliability of academic achievements. Since then, 58 courses have been classified into four types of academic achievements (calculated from the scores of courses), including behavioral and social sciences (11 courses), clinical sciences and skills (24 courses), basic biomedical sciences (17 courses), and scientific method (6 courses), according to the Basic Medical Education WFME Global Standards for Quality Improvement (the 2015 revision) (WFME, 2015). Different academic achievements could represent different kinds of medical abilities. The detailed courses for each academic achievement and the number of students in each course are shown in Supplementary Table 3.

According to the Academic Grading System of Tianjin Medical University, 100 points per course was converted into Grade Point (GP). The detailed conversion criterion is shown in Supplementary Table 4. On this basis, the course credit (C) for each course is taken into account and the Course Grade Point (CGP) was derived from equation [1].


(1) C⁢G⁢P=C×G⁢P

Finally, the GPA for each type of academic achievement was calculated based on equation [2], with *N* representing the number of courses in each type of academic achievement. The GPA for each kind of academic achievement in each student was calculated based on the courses they take.


(2) $$G\,P\,A=\frac{\sum_{1}^{N}C\,G\,P}{\sum_{1}^{N}C}$$

### Personality

Personality data of each participant were evaluated by the Tridimensional Personality Questionnaire (TPQ) and Neuroticism Extraversion Openness (NEO) Personality Inventory (Cloninger, 1987; Cloninger et al., 1991; Caprara et al., 1993). The TPQ is a self-reported personality inventory which evaluates three major personality dimensions, including avoidance of harm (HA), novelty seeking (NS), and reward dependence (RD). Besides, the NEO personality inventory was used to evaluate the big five personality traits which are commonly used models of personality in academic psychology, including extroversion, neuroticism, agreeableness, conscientiousness, and openness to experience. The detailed assessment contents and scientific significance of the two questionnaires were contained in Supplementary Table 5.

### Magnetic resonance imaging data acquisition

Magnetic resonance imaging data were acquired using 3.0-Tesla MR scanners (Discovery MR750, General Electric, Milwaukee, WI, United States). The high-resolution structural T1-weighted images were acquired with the following parameters: repetition time (TR) = 8.16 ms; echo time (TE) = 3.18 ms; inversion time (TI) = 450 ms; matrix = 256 × 256; field of view (FOV) = 256 mm × 256 mm; flip angle (FA) = 12°; slice thickness = 1 mm; and 188 sagittal slices. The resting-state fMRI data were obtained using single shot gradient-echo echo-planar imaging (SS-GRE-EPI): TR = 2,000 ms; TE = 30 ms; matrix = 64 × 64; FOV = 220 mm × 220 mm; FA = 90°; slice thickness = 3 mm; number of slices = 36. During scanning, all participants were asked to stay as motionless as possible, and not to fall asleep.

### fMRI data preprocessing

Statistical Parametric Mapping (SPM12^1^) was used for the resting-state fMRI data preprocessing. The first five volumes with unstable signals were discarded. The collection time delay between slices is corrected by sinc-interpolation so that the collection time of all voxels is consistent within a repetition time. The head movements of each subject were assessed and corrected using rigid body transformations. All subjects satisfied the requirement for head motion (translation < 2 mm and rotation < 2°). The structural images were segmented and coregistered to the Montreal Neurological Institute (MNI) space. The parameters from structural images were used to normalize the fMRI images into the MNI space. The framewise displacement (FD) was also calculated for fMRI images, which indexes volume-to-volume changes in head position. The normalized fMRI images were resampled to 3-mm cubic voxels and smoothed using an 8-mm full-width at half-maximum (FWHM) Gaussian kernel. Spurious variances of the fMRI data were removed by regressing out the average blood oxygenation level-dependent (BOLD) signals of white matter (WM), cerebral spinal fluid (CSF), and 24 head motion parameters. Finally, the fMRI images were filtered using the frequency range of 0.01 to 0.08 Hz.

### ReHo calculation

ReHo and ALFF are both reliable and reproducible parameters of functional MRI to reflect the level of regional functional neural activity. ReHo reflects the coherence of regional brain activity. For a given voxel, ReHo was defined as Kendall’s coefficient of concordance (KCC) of the time series of this voxel with its nearest neighbors (26 voxels) (Zang et al., 2004). ReHo of each grey matter (GM) voxel was calculated based on the normalized fMRI data, and the resulting ReHo map was further standardized, with each individual ReHo map divided by its mean ReHo of all GM voxels. A Gaussian kernel of 8 mm × 8 mm × 8 mm FWHM was used to smooth the data to reduce noise after calculating the ReHo.

### Amplitude of low frequency fluctuation calculation

Amplitude of low frequency fluctuation represents the amplitude of the low-frequency BOLD signal fluctuation. The preprocessed time series were transformed to a frequency domain with a fast Fourier transform (FFT) and the power spectrum was then obtained. Because the power of a given frequency is proportional to the square of the amplitude of this frequency component of the original time series in the time domain, the square root was calculated at each frequency of the power spectrum, and the averaged square root was obtained across 0.01–0.08 Hz at each voxel. This averaged square root was taken as the ALFF. For standardization purposes, the ALFF of each voxel was divided by the global mean ALFF value of each subject.

### Functional connectivity density and strength functional connectivity strength calculation

The FCD and FCS of each voxel were calculated to represent brain function at the connectivity level (Tomasi and Volkow, 2011). A GM mask was used to restrict the voxels in the GM regions with a probability of voxels within the mask belonging to GM > 50%. Pearson’s correlation coefficients were calculated between the BOLD time courses of all pairs of voxels and a whole GM functional connectivity matrix was obtained for each subject. Two voxels with a correlation coefficient of *R* > 0.6 were considered functionally connected for FCD, which was suggested to be the most optimal threshold for calculating FCD (Tomasi and Volkow, 2011). For FCS analysis, to eliminate weak correlations possibly arising from background noise, we restricted our analysis to positive correlations above a threshold of 0.2. The total number of functional connections was defined as the FCD for a voxel and the average strength of functional connections was defined as the FCS. This calculation was performed in each voxel throughout the brain and Fisher’s r-to-z transformation was performed to increase the normality of the distribution. Finally, the FCD and FCS maps were smoothed with an 8 mm × 8 mm × 8 mm Gaussian kernel.

### Grey matter volume calculation

CAT12 toolbox (version r1364^2^) was used for GMV calculation. The structural images were segmented into GM, WM, and CSF after the bias-field inhomogeneity was corrected, and then affine registration to standard space. The diffeomorphic anatomical registration through the exponentiated lie algebra (DARTEL) technique was used in normalization and the normalized images were resampled to a voxel size of 1.5 mm × 1.5 mm × 1.5 mm (Ashburner, 2007). The modulation was performed on the normalized GM images to preserve the absolute volume of the GM tissue. Finally, the images were smoothed with an 8 mm × 8 mm × 8 mm Gaussian kernel.

### Association analysis between personality traits and academic achievements

Linear regression estimate was performed between the scores of HA, NS, and RD calculated from TPQ, five personality scores calculated from the NEO personality inventory and the four types of academic achievements. Specially, age, gender, and education years were considered as nuisance variables and regressed out in the analysis. The β values and *P* values were obtained and the significance threshold was defined as *P* < 0.05.

### Association analysis between brain properties and academic achievements

Statistical parametric mapping [SPM12^(*see text footnote 1*)^] was used to explore which voxels were correlated with academic achievements. Age, gender, education years, and total intracranial volume were regressed out in the association analysis. Multiple comparisons were corrected by a false discovery rate (FDR) method (the threshold was voxel-wise FDR: *q* < 0.05 and a cluster size of at least 50 voxels).

### Multivariate mediation analysis

The PROCESS macro (v3.5) for SPSS was used in multivariate mediation analysis (Hayes, 2013). Only personality traits and brain properties with significant correlations with academic achievements were included in the mediation analysis. On the one hand, the personality score was defined as an independent variable, the brain properties were defined as mediator variables, and the academic achievement was defined as a dependent variable. On the other hand, the brain property was defined as the independent variable, the personalities were defined as mediator variables, and academic achievement was defined as a dependent variable. Age, gender, and education years were regarded as covariates. In multivariate mediation analysis, all indirect effects are estimated in one multiple regression analysis with independent variable and all mediators as predictor variables. We used bootstrapping to assess the significance of the mediation effect. After 5,000 bias-corrected bootstrapping, we estimated the distribution of the indirect effects and calculate their 95% confidence intervals (CI). If zero does not fall between the resulting 95% confidence interval of the bootstrapping method, we confirmed the existence of a significant mediation effect (*P* < 0.05).

## Results

### Academic achievements associated with personality traits

**FIGURE 2:**
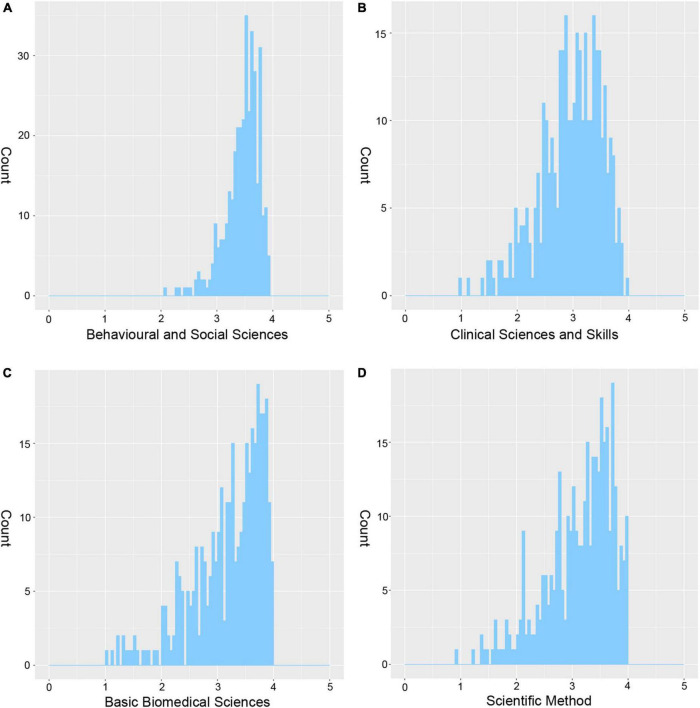
The distribution of grade point average in each type of academic achievements. **(A–D)** The distribution of GPA scores in behavioral and social sciences, clinical sciences and skills, basic biomedical sciences, and scientific method, respectively. The *x*-axis shows the GPA scores and the *y*-axis shows the number of subjects.

The 58 courses were included and divided into four types of academic achievements, followed by the GPA distribution for each of the four types, as shown in Figure 2. In addition, to further validate the robustness of our calculations, we also calculated the GPA based on a “point to point” theory, which means that when the scale of the score was between 60 and 89, the higher the score, the higher GPA available based on linear transformation (Supplementary Table 6). Spearman correlations were performed between the GPA used in our work and the new GPA calculated based on the “point to point” theory, as shown in Supplementary Figure 2, significant correlations were found in all four academic achievements, which further suggests that the GPA we used is robust.

Meanwhile, the distribution of GPA scores for four types of academic achievements in master and bachelor were shown in Supplementary Figure 3. A higher score was found in the master group in each kind of academic achievement and a two-sample *t*-test was performed to make a comparison between the master group and bachelor group (*P*_*Scientific method*_ = 0.04, *P*_*Behavioral and social sciences*_ = 0.04, *P*_*Basic biomedical sciences*_ = 0.02, *P*_*Clinical sciences and skills*_ = 8.92E-5). In addition, we calculated the total score based on the four kinds of academic achievements (the sum of the GPA from four kinds of academic achievements), and the distribution of which was shown in Supplementary Figure 4. Furthermore, Spearman correlations were performed among the four kinds of academic achievements. The *rho* values were shown in Supplementary Table 7, and there were significant correlations among different kinds of academic achievements (*P* < 0.001).

Based on the HA, NS, and RD scores calculated from TPQ, linear regression analyses found that the NS scores showed significantly negative association with all the four types of academic achievements (behavioral and social sciences: *P* = 3.86E-5, β = −3.03; clinical sciences and skills: *P* = 0.02, β = −0.89; basic biomedical sciences: *P* = 1.60E-4, β = −1.31; and scientific method: *P* = 5.85E-4, β = −1.14). Besides, the conscientiousness scores calculated from the NEO personality inventory showed significantly positive association with all the four types of academic achievements (behavioral and social sciences: *P* = 4.59E-4, β = 4.19; clinical sciences and skills: *P* = 7.41E-3, β = 1.73; basic biomedical sciences: *P* = 4.61E-3, β = 1.61; and scientific method: *P* = 1.37E-4, β = 2.06), the results are shown in Tables 1, 2. Besides, the total GPA also showed a significant negative association with the NS scores and showed a significant positive association with the conscientiousness scores, which led to the same conclusion as the four kinds of academic achievements (Supplementary Table 8). These findings informed that NS and conscientiousness personality traits may exert different influences on the academic achievements of medical students.

**TABLE 1:** Correlations between tridimensional personality questionnaire and academic achievements.

	Scientific method		Behavioral and social sciences		Basic biomedical sciences		Clinical sciences and skills	
	*P*	β	*P*	β	*P*	β	*P*	β
NS	**5.85E-04**	**−1.14**	**3.86E-05**	**−3.03**	**1.60E-04**	**−1.31**	**2.48E-02**	**−0.89**
HA	6.23E-01	**−**0.20	1.84E-01	**−**1.20	8.80E-01	0.06	7.24E-01	**−**0.17
RD	1.21E-01	0.41	4.30E-01	0.47	2.22E-01	0.34	1.55E-01	0.46

The β and P values were obtained from the linear regression estimate, and the bold represented the significant correlations.

HA, harm avoidance; NS, novelty seeking; RD, reward dependence.

**TABLE 2:** The association analysis between the neuroticism extraversion openness personality inventory and academic achievements.

	Scientific method		Behavioral and social sciences		Basic biomedical sciences		Clinical sciences and skills	
	*P*	β	*P*	β	*P*	β	*P*	β
Agreeableness	1.54E-01	0.60	7.08E-01	**−**0.35	3.04E-01	0.45	1.71E-01	0.68
Conscientiousness	**1.37E-04**	**2.06**	**4.59E-04**	**4.19**	**4.61E-03**	**1.61**	**7.41E-03**	**1.73**
Extraversion	4.70E-01	0.44	3.40E-01	1.29	5.42E-01	0.39	1.52E-01	1.04
Neuroticism	4.36E-01	0.54	7.11E-01	0.57	8.30E-01	0.16	4.12E-01	0.68
Openness	1.29E-01	0.68	9.39E-01	**−**0.08	1.58E-01	0.66	1.91E-01	**−**0.70

The β and P values were obtained from the linear regression estimate, and the bold represented the significant correlations.

Taking into account the time gap between MRI/personality assessment and academic achievement assessment, we calculated the time gap between the two time points [i.e., time gap (calendar year) = time_*academic assessment*_−time_*MRI/personality assessment*_], with most participants receiving two evaluations within 2 years. The distribution of the time gap was shown in Supplementary Figure 5. Besides, we regarded the time gap as one of the co-variables and re-performed the association analyses. The NS scores were still significantly negatively correlated with four academic achievements, and the conscientiousness scores were still significantly positively correlated with four academic achievements. The results were presented in Supplementary Tables 9, 10.

### Brain properties associated with academic achievements

**FIGURE 3:**
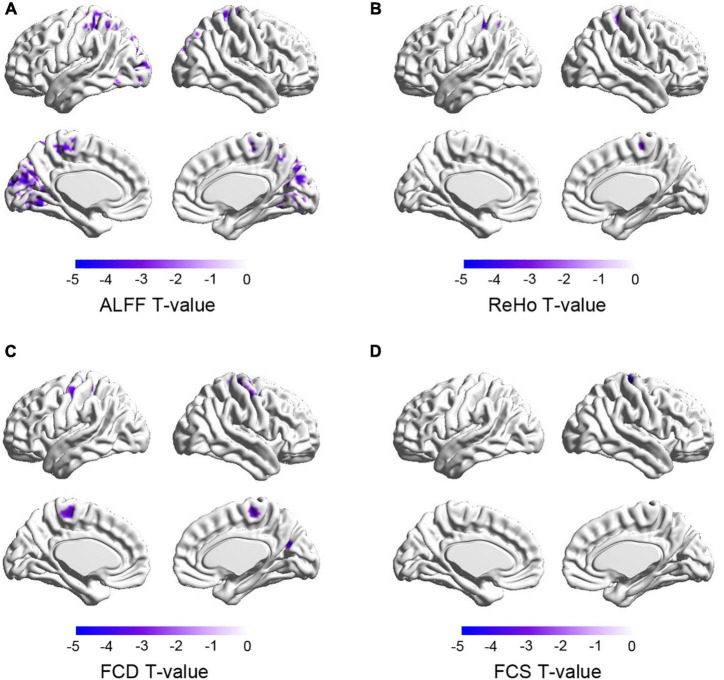
**(A–D)** Functional properties of brain areas correlated with academic achievement in basic biomedical sciences. The color demonstrates the *T*-values of the correlation analyses. ReHo, regional homogeneity; ALFF, amplitude of low frequency fluctuation; FCD, functional connectivity density; FCS, functional connectivity strength.

For each brain imaging measure (i.e., GMV, ReHo, ALFF, FCD, and FCS), voxel-based correlation analyses were performed to explore which brain regions were significantly associated with academic achievements. While we did not find a significant correlation between GMV and academic achievement, we found a significant negative correlation between regional ReHo, ALFF, FCD, and FCS and the GPA scores in basic biomedical sciences. Specifically, the GPA scores of Basic Biomedical Sciences were correlated with ReHo values in the bilateral precentral and postcentral gyri, left superior parietal lobule, right paracentral lobule, and right supplementary motor area; ALFF values in the bilateral occipital gyri, lingual gyri, cuneus, calcarine fissures and surrounding cortices, precentral and postcentral gyri, paracentral lobules, and left superior parietal gyrus; FCD values in the bilateral precentral and postcentral gyri, paracentral lobules, right superior occipital gyrus, cuneus, calcarine fissure and surrounding cortex, and right supplementary motor area; and FCS values in the right precentral gyrus, postcentral gyrus and paracentral lobule (Figure 3). Furthermore, we did not find a significant correlation between each brain imaging measure and total academic achievement.

### Mediation analysis

**FIGURE 4:**
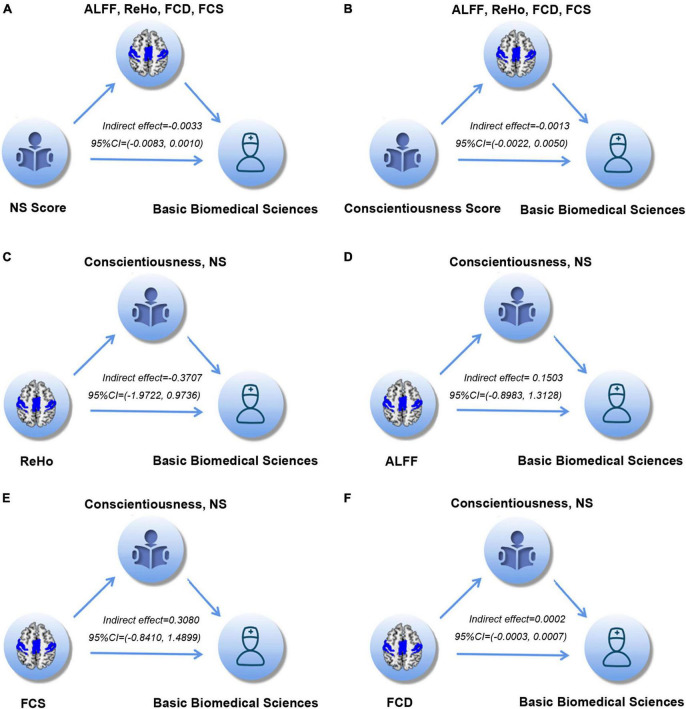
**(A–F)** The multivariate mediation analyses of the associations among personality, brain functional properties and academic achievement of basic biomedical sciences. The indirect effects and their 95%CI were provided. NS, novelty seeking; ReHo, regional homogeneity; ALFF, amplitude of low frequency fluctuation; FCD, functional connectivity density; FCS, functional connectivity strength.

In the mediation analysis, the mediator variables were first defined as the regional ReHo, ALFF, FCD, and FCS values, which were significantly associated with academic achievement in basic biomedical sciences. The values were calculated by the mask which was derived from the significantly correlated brain regions in each brain functional property, respectively. The independent variable was the NS score or the conscientiousness score which showed a significant correlation with academic achievement of basic biomedical sciences, and the dependent variable was defined as the academic achievement of basic biomedical sciences that was correlated with both personality and brain functional properties. The multivariate mediation model aimed to explore whether brain functional properties mediated the associations between personality traits and academic achievements, and we did not find any significant indirect effects (NS: 95% CI of the *indirect effect*,−0.0083 to 0.0010; Conscientiousness: 95% CI of the *indirect effect*, −0.0022 to 0.0050) (Figures 4A,B). Similarly, we changed the independent variable to regional ReHo, ALFF, FCD, and FCS value, respectively, and the mediator variables were defined as the NS score and the conscientiousness score. However, we also did not find any significant indirect effects (ReHo: 95% CI of the *indirect effect*,−1.9722 to 0.9736; ALFF: 95% CI of the *indirect effect*, −0.8983 to 1.3128); FCS: 95% CI of the *indirect effect*, −0.8410 to 1.4899; FCD: 95% CI of the *indirect effect*, −0.0003 to 0.0007 (Figure 4F).

Furthermore, we performed medication analyses based on the brain mask to further validate the mediation results. The mean values of ReHo, ALFF, FCD, and FCS were calculated based on the 90 non-cerebellar regions derived from the Automatic Anatomical Labeling (AAL) mask (Tzourio-Mazoyer et al., 2002). After that, the mediation analysis was performed on each functional property and each brain area. On the one hand, the independent variable was defined as the NS score or the conscientiousness score and the dependent variable was defined as academic achievement of basic biomedical sciences. The mediator variable was defined as the regional ReHo, ALFF, FCD, and FCS value in each brain area, respectively. On the other hand, the independent variable was defined as the regional ReHo, ALFF, FCD, and FCS value in each brain area, respectively. The dependent variable was defined as the academic achievement in basic biomedical sciences. The mediator variable was defined as the NS score or the conscientiousness score. The results showed no significant indirect effects were found (Supplementary Tables 11, 12), further supporting the conclusion that the personality (NS and conscientiousness) and brain properties (functional and connectivity) independently contribute to academic achievements in basic biomedical sciences.

## Discussion

In this study, we investigated the associations between personality traits and brain structural and functional properties of medical students and different types of academic achievements. We found that the NS was negatively correlated with academic achievements, while conscientiousness was positively correlated with academic achievements. In addition, the time gap had little effect on the correlations, and the total GPA score reached the same conclusion, suggesting a stable relationship between medical students’ NS or conscientiousness and academic achievements. Moreover, we found that brain functional (ReHo and ALFF) and connectivity (FCD and FCS) properties rather than structural property (GMV) were negatively correlated with the academic achievement in basic biomedical sciences. However, there was no significant mediation effect among brain functional properties, personality traits (NS and conscientiousness) and academic achievements in basic biomedical sciences. These findings indicate that personality traits (NS and conscientiousness) and brain properties (functional and connectivity) contribute independently to the academic achievements of medical students in basic biomedical sciences, which may help design the promotion plan of medical education.

The tridimensional personality traits were associated with different neurotransmitter systems, such as dopamine-related NS (Benjamin et al., 1996; Ebstein et al., 1996; Stuettgen et al., 2005); 5-transerotonin-associated HA (Hansenne and Ansseau, 1999; Becker et al., 2007); and norepinephrine-associated RD (Garvey et al., 1996). They can predict the adaptive responses of individuals to environmental stimuli. NS reflects the tendency of humans to explore novel and unfamiliar stimuli and environments (Costa et al., 2014), and a higher NS score has been associated with greater impulsivity, sensation seeking, orientation toward independence, openness to experience, risk-taking, and extraversion (Wills et al., 1994; Mallet and Vignoli, 2007; Gordon and Luo, 2011). Consistent with previous studies reporting a negative correlation of NS with overall academic achievements (El Sheikh et al., 2014; Jang and Park, 2016), we found a significant negative correlation of NS with all four kinds of academic performances in medical students, which indicates a stable relationship between NS and academic achievements in medical students. Since NS is a personality trait associated with exploration activities, this negative correlation maybe related to individuals with lower NS scores being more academically oriented and less exposed to outside environmental interference.

Consistent with studies reporting positive correlations between conscientiousness and academic achievements (Vedel, 2014; Marcela, 2015; Ahmady et al., 2019), we also found significant positive correlations between conscientiousness and all four types of academic achievements in medical students. Conscientiousness reflects the overall motivational tendencies (Kanfer and Heggestad, 1997; Martocchio and Judge, 1997), and higher conscientiousness has been associated with more self-efficient, hard-working, and dedicated (Mount and Ba Rrick, 1995; Chen et al., 2001). High conscientious individuals are more willing to succeed and work harder than low conscientious ones (Gellatly, 2013; Kun et al., 2020). It has been reported that motivation and self-control may be the two neural pathways underlying the impacts of conscientiousness on learning (Park et al., 2017; Gao et al., 2021). These findings indicate that individuals with high conscientiousness have a better ability to control, manage, and regulate, which may contribute to better academic performance.

In this study, we found that brain functional (ReHo and ALFF) and connectivity (FCD and FCS) properties rather than structural property (GMV) were correlated with the academic achievement of basic biomedical sciences, indicating that brain functional activity and connectivity have closer relationships with academic achievements than brain structural property of GMV. However, we did not find any significant correlations of brain functional and connectivity properties with other types of academic achievements, suggesting that brain function and connectivity may only selectively affect a specific domain of academic performance in medical students. More importantly, we found negative correlations between the academic achievement of basic biomedical sciences with brain functional and connectivity properties in the sensorimotor areas, including the somatosensory areas, motor areas, and visual areas. These may represent an indirect association between brain and academic performance. Long-term training has been reported to lead to functional and morphological changes in primary motor and somatosensory cortices (Seidler and Carson, 2017). Besides, based on the idea of “Sensorimotor Learning,” during learning, the brain first converts primal sensory input into potential sensory variables, generating potential motor variables related to behavior, and ultimately driving muscle activations (Sohn et al., 2021). Thus, the somatosensory brain areas may play an important role in learning. Furthermore, increased local functional homogeneity was reported to be associated with reduced complexity of information processing and hierarchies of functional integration or separation within the ventral visual stream (Jiang et al., 2015). In addition, the reduction in ReHo was reported to be associated with stressful tasks (Zhang et al., 2019). These may help to explain the negative correlations between academic achievements and functional properties of sensorimotor areas in the brain.

The multivariate mediation analyses showed the brain functional and connectivity properties that were correlated with academic performance did not show any mediation effects on the associations between personality (NS and conscientiousness) and academic achievement of basic biomedical sciences, and the personality (NS and conscientiousness) also did not show any mediation effects on the associations between the brain functional and connectivity properties and academic achievement of basic biomedical sciences. In addition, brain mask-based mediation analyses did not show significant indirect effects. These findings indicate that personality of NS or conscientiousness, as well as brain functional and connectivity properties, independently contribute to academic achievements in basic biomedical sciences for medical students, informing neurological changes, and personality influences that may affect academic performance through independent pathways.

### Limitations

In interpreting our findings, several limitations should be mentioned. First, the degrees of the included subjects were different (including Bachelor’s, Master’s, and Doctor’s degrees), which can lead to bias on degree differences. Besides, while the time gap has little effect on the results, there was a time gap between the time of MRI/personality assessment and the time of academic achievement assessment for each participant. Future studies should therefore also consider these biases.

## Conclusion

In this study, we found a significant correlation between medical students’ personality traits and academic achievement, suggesting that personality assessment may be useful in student counseling and guidance. We also found that brain functional properties correlate with academic performance; however, no significant mediation effect was found among brain functional properties, personality traits (NS and conscientiousness) and academic achievements in basic biomedical sciences, suggesting that personality (NS and conscientiousness) and brain functional properties independently contribute to the academic performance in basic biomedical sciences for medical students.

## Data availability statement

The raw data supporting the conclusions of this article will be made available by the corresponding authors.

## Ethics statement

The studies involving human participants were reviewed and approved by Tianjin Medical University General Hospital. The patients/participants provided their written informed consent to participate in this study.

## Author contributions

YX and CSY designed research and wrote the manuscript. YX, CCY, MS, and JS performed research. YX, CCY, MS, HX, and SW were involved in the assessment. NZ, WQ, FL, HD, JH, LH, XL, and CSY provided guidance and advice. All authors contributed to the article and approved the submitted version.

## Footnotes

1
http://www.fil.ion.ucl.ac.uk/spm

2
http://dbm.neuro.uni-jena.de/cat
