# Supplementary material for: Personality and brain contribute to academic achievements of medical students
Source: Front Neurosci. 2022 Sep 6;16:964904. doi: 10.3389/fnins.2022.964904 (PMC9489117; doi:10.3389/fnins.2022.964904)
Supplement: Supplementary file 1 [file Data_Sheet_1.pdf]

Supplementary Materials

Personality and Brain Contribute to Academic Achievements of Medical Students

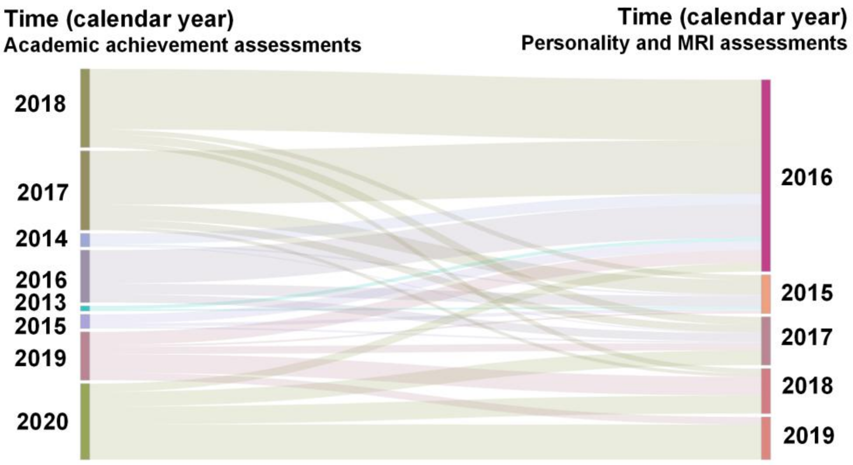

Supplementary Figure 1. The corresponding time (calendar year) of academic achievement assessment and personality/MRI assessment for each subject.

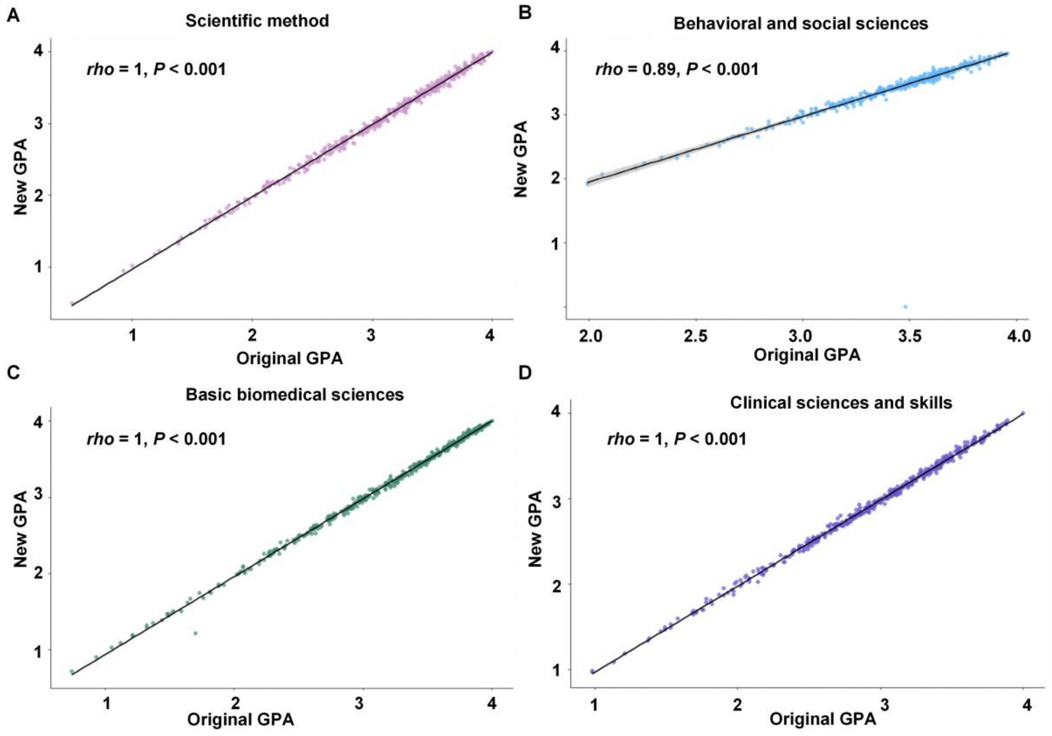

Supplementary Figure 2. The correlation between original GPA and new GPA. The x-axis shows the original GPA scores and the y-axis shows the new GPA scores based on the theory of "point to point".

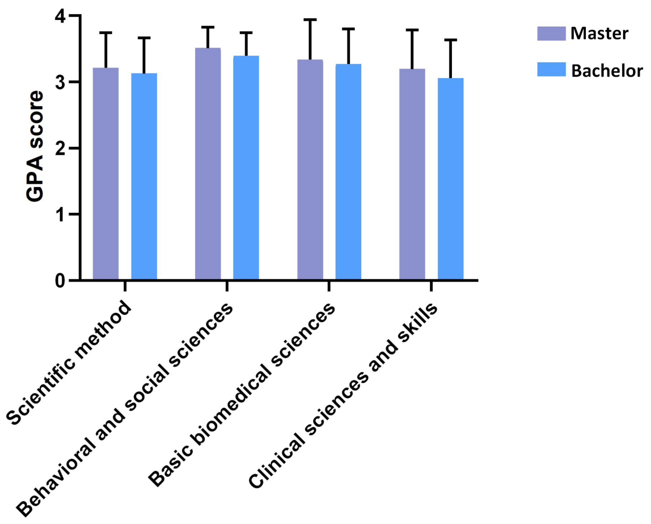

Supplementary Figure 3. The distribution of GPA scores in four kinds of academic achievements in master and bachelor. The purple color represents the GPA scores of the master, the blue color represents the GPA scores of the bachelor.

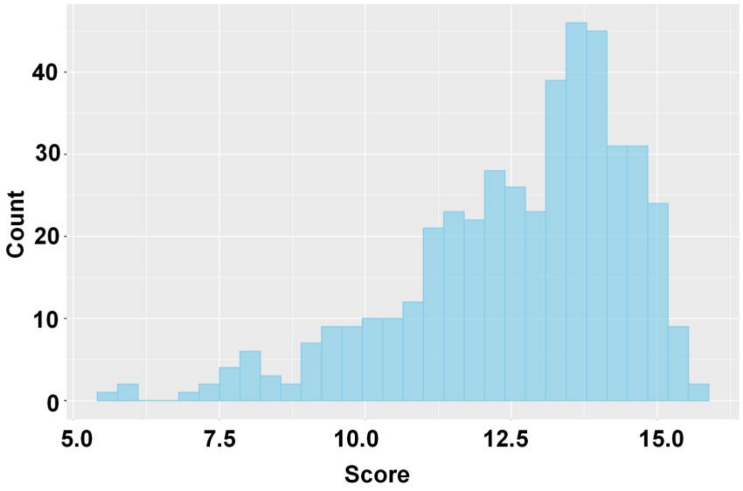

Supplementary Figure 4. The distribution of total GPA score. The x-axis shows the GPA scores and the y-axis shows the number of subjects.

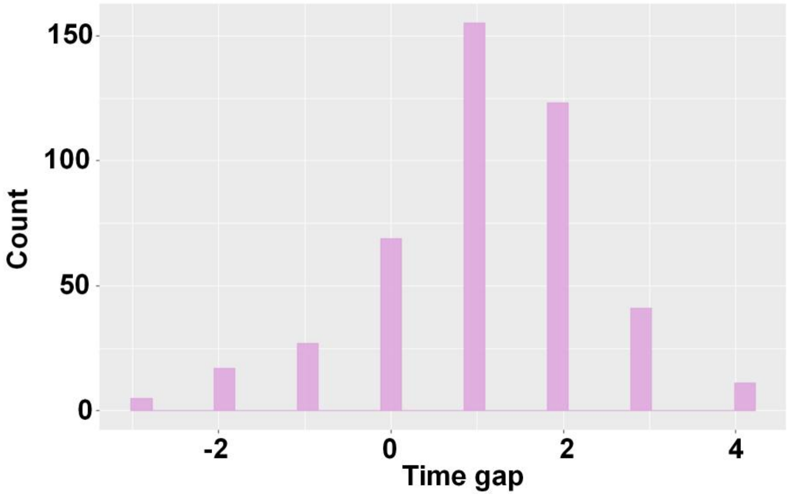

Supplementary Figure 5. The distribution of time gap between academic achievement assessment and personality/MRI assessment. Time gap (calendar year) was evaluated by $time_{\text{academic assessment}}$ - $time_{\text{MRI/personality assessment.}}$

Supplementary Tables

Supplementary Table 1. Inclusion and exclusion criteria for the participants

Category	Item
Inclusion criteria	Age of 18-30 years
	Chinese Han without other ethnic ancestors in recent three generations
	Right-handedness confirmed by the handedness questionnaire
Exclusion criteria	Relatives have participated in this study
	Neuropsychiatric diseases (schizophrenia, anxiety, depression, epilepsy, stroke, tumors, and multiple sclerosis, etc.)
	Consciousness loss for more than 5 minutes
	Brain injury or neurosurgery
	Major physical illnesses (heart disease, hypertension, nephritis, diabetes, malignant tumors, hereditary diseases, etc.)
	Visible brain abnormalities on previous MRI examinations
	Psychiatric disorders in three generation relatives
	Alcohol or drug abuse or dependence
	The total number of cigarettes so far is more than 20
	Currently with any medication (including contraceptives)
	Taking drugs (antipsychotics, mood stabilizers, isoniazid, glucocorticoids, stimulants, etc.) that might affect the brain
	Using sedative hypnotics (benzodiazepines, barbiturates) within one month
	Color blindness or difficulty in color discrimination
	Women in pregnancy or in the menstrual period on the day of the experiment
	Strenuous exercise or consumption of strong tea, caffeine or alcoholic beverages on the day of the experiment
	Without enough sleep (< 7 hours) at the night before the experiment
	Metal implants
	Electronic implants (e.g., pacemakers)
	Severe claustrophobia

Supplementary Table 2. Detailed degree types of subjects and academic achievements period

ID	Personality and MRI assessments			Academic achievement assessments		
	Time (calendar year)	Academic degree	Years of education	Time (calendar year)	Source of score	Years of education
Sub001	2015.11	master	18	2014.07	5 years（2009.09-2014.07）	17
Sub002	2015.10	master	17	2015.07	5 years（2010.09-2015.07）	17
Sub003	2015.11	master	16	2016.07	5 years（2011.09-2016.07）	17
Sub004	2015.11	bachelor	13	2019.07	5 years（2014.09-2019.07）	17
Sub005	2015.11	bachelor	15	2017.07	5 years（2012.09-2017.07）	17
Sub006	2015.11	bachelor	15	2017.07	5 years（2012.09-2017.07）	17
Sub007	2015.11	bachelor	16	2017.07	5 years（2012.09-2017.07）	18
Sub008	2015.11	bachelor	16	2017.07	5 years（2012.09-2017.07）	18
Sub009	2015.11	bachelor	15	2017.07	5 years（2012.09-2017.07）	17
Sub010	2015.11	bachelor	15	2017.07	5 years（2012.09-2017.07）	17
Sub011	2015.12	bachelor	15	2017.07	5 years（2012.09-2017.07）	17
Sub012	2015.12	bachelor	14	2018.07	5 years（2013.09-2018.07）	17
Sub013	2015.12	bachelor	14	2018.07	5 years（2013.09-2018.07）	17
Sub014	2015.12	bachelor	14	2018.07	5 years（2013.09-2018.07）	17
Sub015	2015.12	bachelor	14	2015.07	5 years（2010.09-2015.07）	14
Sub016	2015.12	bachelor	15	2018.07	5 years（2013.09-2018.07）	18
Sub017	2015.12	bachelor	14	2013.07	5 years（2008.09-2013.07）	12
Sub018	2015.12	bachelor	14	2017.07	5 years（2012.09-2017.07）	16
Sub019	2015.12	bachelor	14	2018.07	5 years（2013.09-2018.07）	17
Sub020	2015.12	master	19	2014.07	5 years（2009.09-2014.07）	18
Sub021	2016.01	bachelor	16	2016.07	5 years（2011.09-2016.07）	16
Sub022	2016.01	master	17	2015.07	5 years（2010.09-2015.07）	16
Sub023	2016.01	master	19	2014.07	5 years（2009.09-2014.07）	17
Sub024	2016.01	master	18	2014.07	5 years（2009.09-2014.07）	16
Sub025	2016.01	master	18	2014.07	5 years（2009.09-2014.07）	16
Sub026	2016.01	bachelor	15	2017.07	5 years（2012.09-2017.07）	16
Sub027	2016.06	master	18	2014.07	5 years（2009.09-2014.07）	16
Sub028	2016.01	bachelor	15	2017.07	5 years（2012.09-2017.07）	16
Sub029	2016.01	bachelor	15	2017.07	5 years（2012.09-2017.07）	16
Sub030	2016.01	master	21	2014.07	5 years（2009.09-2014.07）	19
Sub031	2016.01	bachelor	17	2015.07	5 years（2010.09-2015.07）	16
Sub032	2016.02	bachelor	17	2016.07	5 years（2011.09-2016.07）	17
Sub033	2016.03	master	18	2015.07	5 years（2010.09-2015.07）	17
Sub034	2016.03	bachelor	15	2018.07	5 years（2013.09-2018.07）	17
Sub035	2016.03	bachelor	15	2018.07	5 years（2013.09-2018.07）	17
Sub036	2016.03	master	17	2015.07	5 years（2010.09-2015.07）	16
Sub037	2016.03	bachelor	15	2018.07	5 years（2013.09-2018.07）	17

Sub038	2016.03	bachelor	15	2018.07	5 years（2013.09-2018.07）	17
Sub039	2016.03	bachelor	16	2017.07	5 years（2012.09-2017.07）	17
Sub040	2016.03	bachelor	16	2017.07	5 years（2012.09-2017.07）	17
Sub041	2016.07	bachelor	16	2019.07	5 years（2014.09-2019.07）	19
Sub042	2016.03	bachelor	17	2016.07	5 years（2011.09-2016.07）	17
Sub043	2016.03	bachelor	15	2017.07	5 years（2012.09-2017.07）	16
Sub044	2016.03	bachelor	16	2016.07	5 years（2011.09-2016.07）	16
Sub045	2016.03	bachelor	17	2016.07	5 years（2011.09-2016.07）	17
Sub046	2016.04	bachelor	15	2017.07	5 years（2012.09-2017.07）	16
Sub047	2016.03	bachelor	16	2017.07	5 years（2012.09-2017.07）	17
Sub048	2016.04	bachelor	15	2017.07	5 years（2012.09-2017.07）	16
Sub049	2016.04	bachelor	16	2016.07	5 years（2011.09-2016.07）	16
Sub050	2016.04	master	19	2015.07	5 years（2010.09-2015.07）	18
Sub051	2016.04	bachelor	17	2016.07	5 years（2011.09-2016.07）	17
Sub052	2016.04	master	17	2014.07	5 years（2009.09-2014.07）	15
Sub053	2016.04	master	17	2014.07	5 years（2009.09-2014.07）	15
Sub054	2016.04	bachelor	16	2017.07	5 years（2012.09-2017.07）	17
Sub055	2016.05	bachelor	15	2018.07	5 years（2013.09-2018.07）	17
Sub056	2016.05	bachelor	15	2018.07	5 years（2013.09-2018.07）	17
Sub057	2016.05	bachelor	17	2016.07	5 years（2011.09-2016.07）	17
Sub058	2016.04	bachelor	15	2018.07	5 years（2013.09-2018.07）	17
Sub059	2016.05	bachelor	18	2016.07	5 years（2011.09-2016.07）	18
Sub060	2016.05	bachelor	15	2018.07	5 years（2013.09-2018.07）	17
Sub061	2016.05	bachelor	15	2018.07	5 years（2013.09-2018.07）	17
Sub062	2016.05	bachelor	15	2018.07	5 years（2013.09-2018.07）	17
Sub063	2016.05	bachelor	15	2018.07	5 years（2013.09-2018.07）	17
Sub064	2016.05	bachelor	15	2018.07	5 years（2013.09-2018.07）	17
Sub065	2016.05	bachelor	15	2018.07	5 years（2013.09-2018.07）	17
Sub066	2016.05	bachelor	15	2018.07	5 years（2013.09-2018.07）	17
Sub067	2016.05	bachelor	15	2018.07	5 years（2013.09-2018.07）	17
Sub068	2016.05	bachelor	15	2018.07	5 years（2013.09-2018.07）	17
Sub069	2016.05	bachelor	15	2018.07	5 years（2013.09-2018.07）	17
Sub070	2016.05	bachelor	15	2018.07	5 years（2013.09-2018.07）	17
Sub071	2016.05	bachelor	15	2018.07	5 years（2013.09-2018.07）	17
Sub072	2016.05	bachelor	15	2018.07	5 years（2013.09-2018.07）	17
Sub073	2016.05	master	19	2014.07	5 years（2009.09-2014.07）	17
Sub074	2016.06	master	19	2014.07	5 years（2009.09-2014.07）	17
Sub075	2016.06	master	18	2015.07	5 years（2010.09-2015.07）	17
Sub076	2016.06	bachelor	14	2019.07	5 years（2014.09-2019.07）	17
Sub077	2016.06	bachelor	14	2019.07	5 years（2014.09-2019.07）	17
Sub078	2016.06	bachelor	14	2019.07	5 years（2014.09-2019.07）	17
Sub079	2016.06	bachelor	16	2017.07	5 years（2012.09-2017.07）	17
Sub080	2016.07	master	18	2015.07	5 years（2010.09-2015.07）	17
Sub081	2016.07	master	17	2016.07	5 years（2011.09-2016.07）	17

Sub082	2016.07	bachelor	16	2017.07	5 years（2012.09-2017.07）	17
Sub083	2016.07	master	15	2018.07	5 years（2013.09-2018.07）	17
Sub084	2016.07	bachelor	16	2017.07	5 years（2012.09-2017.07）	17
Sub085	2016.07	bachelor	16	2017.07	5 years（2012.09-2017.07）	17
Sub086	2016.07	bachelor	17	2017.07	5 years（2012.09-2017.07）	18
Sub087	2016.07	bachelor	16	2017.07	5 years（2012.09-2017.07）	17
Sub088	2016.07	bachelor	16	2017.07	5 years（2012.09-2017.07）	17
Sub089	2016.08	bachelor	17	2017.07	5 years（2012.09-2017.07）	18
Sub090	2016.07	bachelor	16	2017.07	5 years（2012.09-2017.07）	17
Sub091	2016.07	bachelor	16	2017.07	5 years（2012.09-2017.07）	17
Sub092	2016.07	bachelor	16	2017.07	5 years（2012.09-2017.07）	17
Sub093	2016.07	bachelor	15	2017.07	5 years（2012.09-2017.07）	16
Sub094	2016.07	bachelor	17	2017.07	5 years（2012.09-2017.07）	18
Sub095	2016.07	bachelor	16	2017.07	5 years（2012.09-2017.07）	17
Sub096	2016.08	bachelor	17	2017.07	5 years（2012.09-2017.07）	18
Sub097	2016.08	bachelor	17	2017.07	5 years（2012.09-2017.07）	18
Sub098	2016.08	bachelor	16	2017.07	5 years（2012.09-2017.07）	17
Sub099	2016.08	bachelor	16	2017.07	5 years（2012.09-2017.07）	17
Sub100	2016.08	bachelor	16	2017.07	5 years（2012.09-2017.07）	17
Sub101	2016.08	bachelor	16	2017.07	5 years（2012.09-2017.07）	17
Sub102	2016.08	bachelor	16	2017.07	5 years（2012.09-2017.07）	17
Sub103	2016.08	bachelor	16	2017.07	5 years（2012.09-2017.07）	17
Sub104	2016.08	master	17	2016.07	5 years（2011.09-2016.07）	17
Sub105	2016.08	bachelor	17	2017.07	5 years（2012.09-2017.07）	18
Sub106	2016.08	bachelor	16	2017.07	5 years（2012.09-2017.07）	17
Sub107	2016.08	bachelor	16	2017.07	5 years（2012.09-2017.07）	17
Sub108	2016.08	bachelor	13	2019.07	5 years（2014.09-2019.07）	16
Sub109	2016.09	master	15	2018.07	5 years（2013.09-2018.07）	17
Sub110	2016.09	master	17	2017.07	5 years（2012.09-2017.07）	18
Sub111	2016.10	bachelor	13	2020.07	5 years（2015.09-2020.07）	17
Sub112	2016.09	bachelor	15	2018.07	5 years（2013.09-2018.07）	17
Sub113	2016.09	bachelor	14	2018.07	5 years（2013.09-2018.07）	16
Sub114	2016.09	bachelor	16	2018.07	5 years（2013.09-2018.07）	18
Sub115	2016.09	bachelor	15	2018.07	5 years（2013.09-2018.07）	17
Sub116	2016.09	bachelor	16	2018.07	5 years（2013.09-2018.07）	18
Sub117	2016.09	bachelor	15	2018.07	5 years（2013.09-2018.07）	17
Sub118	2016.09	bachelor	14	2018.07	5 years（2013.09-2018.07）	16
Sub119	2016.09	bachelor	16	2017.07	5 years（2012.09-2017.07）	17
Sub120	2016.09	bachelor	16	2018.07	5 years（2013.09-2018.07）	18
Sub121	2016.10	bachelor	14	2019.07	5 years（2014.09-2019.07）	17
Sub122	2016.10	bachelor	16	2017.07	5 years（2012.09-2017.07）	17
Sub123	2016.10	bachelor	15	2019.07	5 years（2014.09-2019.07）	18
Sub124	2016.10	bachelor	14	2018.07	5 years（2013.09-2018.07）	16
Sub125	2016.10	bachelor	14	2019.07	5 years（2014.09-2019.07）	17

Sub126	2016.10	bachelor	14	2018.07	5 years（2013.09-2018.07）	16
Sub127	2016.10	bachelor	13	2020.07	5 years（2015.09-2020.07）	17
Sub128	2016.10	bachelor	14	2019.07	5 years（2014.09-2019.07）	17
Sub129	2016.10	bachelor	13	2020.07	5 years（2015.09-2020.07）	17
Sub130	2016.10	bachelor	15	2018.07	5 years（2013.09-2018.07）	17
Sub131	2016.10	bachelor	12	2020.07	4 years（2016.09-2020.07）	16
Sub132	2016.10	bachelor	13	2020.07	5 years（2015.09-2020.07）	17
Sub133	2016.11	bachelor	15	2017.07	5 years（2012.09-2017.07）	16
Sub134	2016.12	master	17	2016.07	5 years（2011.09-2016.07）	17
Sub135	2016.12	master	17	2016.07	5 years（2011.09-2016.07）	17
Sub136	2017.01	bachelor	15	2017.07	5 years（2012.09-2017.07）	15
Sub137	2017.02	bachelor	14	2019.07	5 years（2014.09-2019.07）	16
Sub138	2017.02	bachelor	14	2019.07	5 years（2014.09-2019.07）	16
Sub139	2017.02	master	19	2015.07	5 years（2010.09-2015.07）	17
Sub140	2017.03	bachelor	13	2020.07	4 years（2016.09-2020.07）	16
Sub141	2017.03	bachelor	15	2019.07	5 years（2014.09-2019.07）	17
Sub142	2017.04	bachelor	14	2020.07	5 years（2015.09-2020.07）	17
Sub143	2017.05	bachelor	13	2020.07	4 years（2016.09-2020.07）	16
Sub144	2017.04	bachelor	13	2020.07	5 years（2015.09-2020.07）	16
Sub145	2017.05	bachelor	14	2020.07	5 years（2015.09-2020.07）	17
Sub146	2017.05	bachelor	15	2019.07	5 years（2014.09-2019.07）	17
Sub147	2017.05	bachelor	17	2017.07	5 years（2012.09-2017.07）	17
Sub148	2017.05	bachelor	13	2020.07	4 years（2016.09-2020.07）	16
Sub149	2017.06	bachelor	16	2018.07	5 years（2013.09-2018.07）	17
Sub150	2017.06	bachelor	16	2018.07	5 years（2013.09-2018.07）	17
Sub151	2017.06	bachelor	13	2020.07	5 years（2015.09-2020.07）	16
Sub152	2017.06	bachelor	15	2019.07	5 years（2014.09-2019.07）	17
Sub153	2017.06	bachelor	16	2019.07	5 years（2014.09-2019.07）	18
Sub154	2017.06	bachelor	13	2020.07	4 years（2016.09-2020.07）	16
Sub155	2017.06	bachelor	14	2020.07	5 years（2015.09-2020.07）	17
Sub156	2017.06	bachelor	15	2019.07	5 years（2014.09-2019.07）	17
Sub157	2017.06	bachelor	14	2020.07	5 years（2015.09-2020.07）	17
Sub158	2017.06	bachelor	14	2019.07	5 years（2014.09-2019.07）	16
Sub159	2017.06	bachelor	15	2019.07	5 years（2014.09-2019.07）	17
Sub160	2017.06	bachelor	13	2020.07	4 years（2016.09-2020.07）	16
Sub161	2017.06	bachelor	14	2020.07	5 years（2015.09-2020.07）	17
Sub162	2017.06	bachelor	14	2020.07	5 years（2015.09-2020.07）	17
Sub163	2017.06	bachelor	15	2020.07	5 years（2015.09-2020.07）	18
Sub164	2017.06	bachelor	14	2020.07	5 years（2015.09-2020.07）	17
Sub165	2017.07	bachelor	13	2020.07	4 years（2016.09-2020.07）	16
Sub166	2017.07	bachelor	16	2018.07	5 years（2013.09-2018.07）	17
Sub167	2017.07	bachelor	17	2018.07	5 years（2013.09-2018.07）	18
Sub168	2017.07	master	17	2016.07	5 years（2011.09-2016.07）	16
Sub169	2017.07	bachelor	16	2018.07	5 years（2013.09-2018.07）	17

Sub170	2017.07	bachelor	16	2018.07	5 years（2013.09-2018.07）	17
Sub171	2017.08	master	18	2016.07	5 years（2011.09-2016.07）	17
Sub172	2017.08	bachelor	17	2018.07	5 years（2013.09-2018.07）	18
Sub173	2017.08	bachelor	16	2018.07	5 years（2013.09-2018.07）	17
Sub174	2017.09	master	17	2016.07	5 years（2011.09-2016.07）	16
Sub175	2017.09	bachelor	15	2020.07	5 years（2015.09-2020.07）	18
Sub176	2017.09	master	18	2016.07	5 years（2011.09-2016.07）	17
Sub177	2017.09	master	20	2016.07	5 years（2011.09-2016.07）	19
Sub178	2017.09	master	17	2017.07	5 years（2012.09-2017.07）	17
Sub179	2017.09	master	18	2016.07	5 years（2011.09-2016.07）	17
Sub180	2017.09	master	18	2016.07	5 years（2011.09-2016.07）	17
Sub181	2017.09	master	18	2016.07	5 years（2011.09-2016.07）	17
Sub182	2017.09	master	17	2017.07	5 years（2012.09-2017.07）	17
Sub183	2017.09	master	17	2017.07	5 years（2012.09-2017.07）	17
Sub184	2017.10	doctor	20	2014.07	5 years（2009.09-2014.07）	17
Sub185	2017.10	doctor	20	2015.07	5 years（2010.09-2015.07）	18
Sub186	2017.10	master	19	2016.07	5 years（2011.09-2016.07）	18
Sub187	2017.10	master	17	2016.07	5 years（2011.09-2016.07）	16
Sub188	2017.11	master	17	2017.07	5 years（2012.09-2017.07）	17
Sub189	2017.11	bachelor	13	2020.07	4 years（2016.09-2020.07）	16
Sub190	2017.12	bachelor	15	2018.07	5 years（2013.09-2018.07）	16
Sub191	2017.12	bachelor	16	2018.07	5 years（2013.09-2018.07）	17
Sub192	2017.12	master	18	2017.07	5 years（2012.09-2017.07）	18
Sub193	2018.01	bachelor	15	2019.07	5 years（2014.09-2019.07）	16
Sub194	2018.01	bachelor	15	2019.07	5 years（2014.09-2019.07）	16
Sub195	2018.01	bachelor	12	2020.07	3 years（2017.09-2020.07）	14
Sub196	2018.01	bachelor	15	2019.07	5 years（2014.09-2019.07）	16
Sub197	2018.01	bachelor	15	2019.07	5 years（2014.09-2019.07）	16
Sub198	2018.01	bachelor	15	2019.07	5 years（2014.09-2019.07）	16
Sub199	2018.01	bachelor	16	2019.07	5 years（2014.09-2019.07）	17
Sub200	2018.01	bachelor	13	2020.07	3 years（2017.09-2020.07）	15
Sub201	2018.01	bachelor	12	2020.07	3 years（2017.09-2020.07）	14
Sub202	2018.01	bachelor	14	2020.07	5 years（2015.09-2020.07）	16
Sub203	2018.01	bachelor	15	2019.07	5 years（2014.09-2019.07）	16
Sub204	2018.02	bachelor	15	2019.07	5 years（2014.09-2019.07）	16
Sub205	2018.03	bachelor	14	2020.07	4 years（2016.09-2020.07）	16
Sub206	2018.03	bachelor	16	2018.07	5 years（2013.09-2018.07）	16
Sub207	2018.03	bachelor	15	2018.07	5 years（2013.09-2018.07）	15
Sub208	2018.03	bachelor	17	2018.07	5 years（2013.09-2018.07）	17
Sub209	2018.03	bachelor	19	2019.07	5 years（2014.09-2019.07）	20
Sub210	2018.03	bachelor	19	2018.07	5 years（2013.09-2018.07）	19
Sub211	2018.03	bachelor	13	2020.07	3 years（2017.09-2020.07）	15
Sub212	2018.03	bachelor	14	2020.07	5 years（2015.09-2020.07）	16
Sub213	2018.03	bachelor	14	2020.07	5 years（2015.09-2020.07）	16

Sub214	2018.03	bachelor	16	2019.07	5 years（2014.09-2019.07）	17
Sub215	2018.03	bachelor	15	2020.07	5 years（2015.09-2020.07）	17
Sub216	2018.04	bachelor	14	2020.07	4 years（2016.09-2020.07）	16
Sub217	2018.04	bachelor	16	2019.07	5 years（2014.09-2019.07）	17
Sub218	2018.04	bachelor	15	2019.07	5 years（2014.09-2019.07）	16
Sub219	2018.04	bachelor	15	2020.07	5 years（2015.09-2020.07）	17
Sub220	2018.04	bachelor	15	2020.07	5 years（2015.09-2020.07）	17
Sub221	2018.04	bachelor	16	2019.07	5 years（2014.09-2019.07）	17
Sub222	2018.04	bachelor	16	2019.07	5 years（2014.09-2019.07）	17
Sub223	2018.04	bachelor	16	2019.07	5 years（2014.09-2019.07）	17
Sub224	2018.04	bachelor	16	2019.07	5 years（2014.09-2019.07）	17
Sub225	2018.04	bachelor	15	2020.07	5 years（2015.09-2020.07）	17
Sub226	2018.04	bachelor	16	2019.07	5 years（2014.09-2019.07）	17
Sub227	2018.04	bachelor	15	2019.07	5 years（2014.09-2019.07）	16
Sub228	2018.04	bachelor	16	2019.07	5 years（2014.09-2019.07）	17
Sub229	2018.04	bachelor	14	2020.07	4 years（2016.09-2020.07）	16
Sub230	2018.04	bachelor	14	2020.07	4 years（2016.09-2020.07）	16
Sub231	2018.04	bachelor	14	2020.07	4 years（2016.09-2020.07）	16
Sub232	2018.04	bachelor	16	2019.07	5 years（2014.09-2019.07）	17
Sub233	2018.09	master	18	2017.07	5 years（2012.09-2017.07）	17
Sub234	2018.09	master	18	2017.07	5 years（2012.09-2017.07）	17
Sub235	2018.09	master	18	2017.07	5 years（2012.09-2017.07）	17
Sub236	2018.09	bachelor	16	2019.07	5 years（2014.09-2019.07）	17
Sub237	2018.09	bachelor	16	2019.07	5 years（2014.09-2019.07）	17
Sub238	2018.09	bachelor	16	2019.07	5 years（2014.09-2019.07）	17
Sub239	2018.10	master	18	2017.07	5 years（2012.09-2017.07）	17
Sub240	2018.10	bachelor	13	2020.07	3 years（2017.09-2020.07）	15
Sub241	2018.10	bachelor	13	2020.07	3 years（2017.09-2020.07）	15
Sub242	2018.10	bachelor	14	2020.07	3 years（2017.09-2020.07）	16
Sub243	2018.10	bachelor	14	2018.07	5 years（2013.09-2018.07）	14
Sub244	2019.03	bachelor	14	2020.07	3 years（2017.09-2020.07）	15
Sub245	2018.12	bachelor	12	2020.07	3 years（2017.09-2020.07）	14
Sub246	2018.12	master	17	2018.07	5 years（2013.09-2018.07）	17
Sub247	2018.12	bachelor	13	2020.07	3 years（2017.09-2020.07）	15
Sub248	2018.12	bachelor	14	2020.07	3 years（2017.09-2020.07）	16
Sub249	2019.01	bachelor	14	2020.07	3 years（2017.09-2020.07）	15
Sub250	2019.01	bachelor	14	2020.07	3 years（2017.09-2020.07）	15
Sub251	2019.01	bachelor	15	2020.07	5 years（2015.09-2020.07）	16
Sub252	2019.01	bachelor	15	2020.07	5 years（2015.09-2020.07）	16
Sub253	2019.01	bachelor	13	2020.07	3 years（2017.09-2020.07）	14
Sub254	2019.01	bachelor	13	2020.07	3 years（2017.09-2020.07）	14
Sub255	2019.01	bachelor	13	2020.07	3 years（2017.09-2020.07）	14
Sub256	2019.01	bachelor	14	2020.07	4 years（2016.09-2020.07）	15
Sub257	2019.01	bachelor	14	2020.07	4 years（2016.09-2020.07）	15

Sub258	2019.01	bachelor	16	2020.07	5 years（2015.09-2020.07）	17
Sub259	2019.01	bachelor	14	2020.07	5 years（2015.09-2020.07）	15
Sub260	2019.01	bachelor	16	2020.07	5 years（2015.09-2020.07）	17
Sub261	2019.01	bachelor	14	2020.07	4 years（2016.09-2020.07）	15
Sub262	2019.01	bachelor	14	2020.07	4 years（2016.09-2020.07）	15
Sub263	2019.01	bachelor	14	2020.07	4 years（2016.09-2020.07）	15
Sub264	2019.01	bachelor	15	2020.07	5 years（2015.09-2020.07）	16
Sub265	2019.01	bachelor	16	2019.07	5 years（2014.09-2019.07）	16
Sub266	2019.01	bachelor	16	2020.07	5 years（2015.09-2020.07）	17
Sub267	2019.01	bachelor	17	2019.07	5 years（2014.09-2019.07）	17
Sub268	2019.01	bachelor	16	2019.07	5 years（2014.09-2019.07）	16
Sub269	2019.01	bachelor	16	2019.07	5 years（2014.09-2019.07）	16
Sub270	2019.01	bachelor	13	2020.07	3 years（2017.09-2020.07）	14
Sub271	2019.03	bachelor	16	2020.07	3 years（2017.09-2020.07）	17
Sub272	2019.03	bachelor	14	2020.07	4 years（2016.09-2020.07）	15
Sub273	2019.03	bachelor	15	2020.07	5 years（2015.09-2020.07）	16
Sub274	2019.03	bachelor	13	2020.07	3 years（2017.09-2020.07）	14
Sub275	2019.03	bachelor	14	2020.07	3 years（2017.09-2020.07）	15
Sub276	2019.03	bachelor	13	2020.07	3 years（2017.09-2020.07）	14
Sub277	2019.03	bachelor	15	2020.07	4 years（2016.09-2020.07）	16
Sub278	2019.03	bachelor	14	2020.07	4 years（2016.09-2020.07）	15
Sub279	2019.03	bachelor	14	2020.07	4 years（2016.09-2020.07）	15
Sub280	2019.03	bachelor	13	2020.07	3 years（2017.09-2020.07）	14
Sub281	2019.03	bachelor	14	2020.07	4 years（2016.09-2020.07）	15
Sub282	2019.03	bachelor	16	2020.07	5 years（2015.09-2020.07）	17
Sub283	2019.03	bachelor	15	2020.07	5 years（2015.09-2020.07）	16
Sub284	2019.03	bachelor	16	2020.07	5 years（2015.09-2020.07）	17
Sub285	2019.03	bachelor	14	2020.07	3 years（2017.09-2020.07）	15
Sub286	2019.03	bachelor	13	2020.07	3 years（2017.09-2020.07）	14
Sub287	2019.03	bachelor	15	2020.07	5 years（2015.09-2020.07）	16
Sub288	2019.03	bachelor	15	2020.07	5 years（2015.09-2020.07）	16
Sub289	2019.03	bachelor	16	2020.07	5 years（2015.09-2020.07）	17
Sub290	2019.03	bachelor	17	2019.07	5 years（2014.09-2019.07）	17
Sub291	2019.03	bachelor	17	2019.07	5 years（2014.09-2019.07）	17
Sub292	2019.03	bachelor	17	2019.07	5 years（2014.09-2019.07）	17
Sub293	2019.03	bachelor	17	2019.07	5 years（2014.09-2019.07）	17
Sub294	2019.03	bachelor	13	2020.07	3 years（2017.09-2020.07）	14
Sub295	2019.03	bachelor	15	2019.07	5 years（2014.09-2019.07）	15
Sub296	2019.03	bachelor	14	2020.07	4 years（2016.09-2020.07）	15
Sub297	2019.03	bachelor	15	2020.07	4 years（2016.09-2020.07）	16
Sub298	2019.03	bachelor	14	2020.07	4 years（2016.09-2020.07）	15
Sub299	2019.03	bachelor	15	2020.07	4 years（2016.09-2020.07）	16
Sub300	2015.11	bachelor	15	2017.07	5 years（2012.09-2017.07）	17
Sub301	2015.11	bachelor	16	2016.07	5 years（2011.09-2016.07）	17

Sub302	2015.11	bachelor	16	2016.07	5 years（2011.09-2016.07）	17
Sub303	2015.11	bachelor	15	2017.07	5 years（2012.09-2017.07）	17
Sub304	2015.11	bachelor	16	2016.07	5 years（2011.09-2016.07）	17
Sub305	2015.11	bachelor	16	2016.07	5 years（2011.09-2016.07）	17
Sub306	2015.11	bachelor	16	2016.07	5 years（2011.09-2016.07）	17
Sub307	2015.11	bachelor	16	2016.07	5 years（2011.09-2016.07）	17
Sub308	2015.11	bachelor	17	2017.07	5 years（2012.09-2017.07）	19
Sub309	2015.11	bachelor	17	2016.07	5 years（2011.09-2016.07）	18
Sub310	2015.11	bachelor	17	2015.07	5 years（2010.09-2015.07）	17
Sub311	2015.11	bachelor	15	2017.07	5 years（2012.09-2017.07）	17
Sub312	2015.11	bachelor	16	2016.07	5 years（2011.09-2016.07）	17
Sub313	2015.11	bachelor	14	2017.07	5 years（2012.09-2017.07）	16
Sub314	2015.11	bachelor	15	2019.07	5 years（2014.09-2019.07）	18
Sub315	2015.12	bachelor	15	2017.07	5 years（2012.09-2017.07）	17
Sub316	2015.12	bachelor	17	2017.07	5 years（2012.09-2017.07）	19
Sub317	2015.12	master	19	2013.07	5 years（2008.09-2013.07）	17
Sub318	2015.12	bachelor	18	2016.07	5 years（2011.09-2016.07）	19
Sub319	2015.12	bachelor	15	2017.07	5 years（2012.09-2017.07）	17
Sub320	2015.12	bachelor	15	2017.07	5 years（2012.09-2017.07）	17
Sub321	2015.12	bachelor	15	2018.07	5 years（2013.09-2018.07）	18
Sub322	2015.12	bachelor	17	2016.07	5 years（2011.09-2016.07）	18
Sub323	2015.12	bachelor	17	2016.07	5 years（2011.09-2016.07）	18
Sub324	2015.12	bachelor	17	2016.07	5 years（2011.09-2016.07）	18
Sub325	2015.12	master	17	2015.07	5 years（2010.09-2015.07）	17
Sub326	2015.12	bachelor	14	2017.07	5 years（2012.09-2017.07）	16
Sub327	2016.01	master	19	2013.07	5 years（2008.09-2013.07）	16
Sub328	2016.01	bachelor	16	2017.07	5 years（2012.09-2017.07）	17
Sub329	2016.01	master	17	2013.07	5 years（2008.09-2013.07）	14
Sub330	2016.01	bachelor	14	2017.07	5 years（2012.09-2017.07）	15
Sub331	2016.02	bachelor	16	2016.07	5 years（2011.09-2016.07）	16
Sub332	2016.03	bachelor	14	2018.07	5 years（2013.09-2018.07）	16
Sub333	2016.03	bachelor	14	2018.07	5 years（2013.09-2018.07）	16
Sub334	2016.03	bachelor	19	2016.07	5 years（2011.09-2016.07）	19
Sub335	2016.03	master	14	2018.07	5 years（2013.09-2018.07）	16
Sub336	2016.03	bachelor	16	2018.07	5 years（2013.09-2018.07）	18
Sub337	2016.03	master	19	2014.07	5 years（2009.09-2014.07）	17
Sub338	2016.03	bachelor	14	2018.07	5 years（2013.09-2018.07）	16
Sub339	2016.03	bachelor	15	2017.07	5 years（2012.09-2017.07）	16
Sub340	2016.03	bachelor	16	2017.07	5 years（2012.09-2017.07）	17
Sub341	2016.03	bachelor	16	2017.07	5 years（2012.09-2017.07）	17
Sub342	2016.04	bachelor	15	2017.07	5 years（2012.09-2017.07）	16
Sub343	2016.04	bachelor	15	2017.07	5 years（2012.09-2017.07）	16
Sub344	2016.04	master	19	2015.07	5 years（2010.09-2015.07）	18
Sub345	2016.04	bachelor	16	2016.07	5 years（2011.09-2016.07）	16

Sub346	2016.04	bachelor	15	2017.07	5 years（2012.09-2017.07）	16
Sub347	2016.04	bachelor	15	2018.07	5 years（2013.09-2018.07）	17
Sub348	2016.04	master	15	2016.07	5 years（2011.09-2016.07）	15
Sub349	2016.04	bachelor	17	2016.07	5 years（2011.09-2016.07）	17
Sub350	2016.04	bachelor	17	2016.07	5 years（2011.09-2016.07）	17
Sub351	2016.04	bachelor	16	2016.07	5 years（2011.09-2016.07）	16
Sub352	2016.04	bachelor	16	2017.07	5 years（2012.09-2017.07）	17
Sub353	2016.04	bachelor	15	2017.07	5 years（2012.09-2017.07）	16
Sub354	2016.04	bachelor	15	2017.07	5 years（2012.09-2017.07）	16
Sub355	2016.04	bachelor	16	2017.07	5 years（2012.09-2017.07）	17
Sub356	2016.04	bachelor	14	2018.07	5 years（2013.09-2018.07）	16
Sub357	2016.04	bachelor	15	2018.07	5 years（2013.09-2018.07）	17
Sub358	2016.04	bachelor	15	2018.07	5 years（2013.09-2018.07）	17
Sub359	2016.04	bachelor	15	2018.07	5 years（2013.09-2018.07）	17
Sub360	2016.04	bachelor	15	2018.07	5 years（2013.09-2018.07）	17
Sub361	2016.04	bachelor	15	2018.07	5 years（2013.09-2018.07）	17
Sub362	2016.04	bachelor	15	2018.07	5 years（2013.09-2018.07）	17
Sub363	2016.05	bachelor	15	2018.07	5 years（2013.09-2018.07）	17
Sub364	2016.05	bachelor	16	2018.07	5 years（2013.09-2018.07）	18
Sub365	2016.05	bachelor	14	2018.07	5 years（2013.09-2018.07）	16
Sub366	2016.05	bachelor	17	2018.07	5 years（2013.09-2018.07）	19
Sub367	2016.05	bachelor	17	2016.07	5 years（2011.09-2016.07）	17
Sub368	2016.05	bachelor	14	2018.07	5 years（2013.09-2018.07）	16
Sub369	2016.05	bachelor	16	2016.07	5 years（2011.09-2016.07）	16
Sub370	2016.05	bachelor	16	2017.07	5 years（2012.09-2017.07）	17
Sub371	2016.05	bachelor	14	2018.07	5 years（2013.09-2018.07）	16
Sub372	2016.05	bachelor	15	2018.07	5 years（2013.09-2018.07）	17
Sub373	2016.05	bachelor	15	2018.07	5 years（2013.09-2018.07）	17
Sub374	2016.05	bachelor	15	2018.07	5 years（2013.09-2018.07）	17
Sub375	2016.05	bachelor	15	2017.07	5 years（2012.09-2017.07）	16
Sub376	2016.05	master	19	2014.07	5 years（2009.09-2014.07）	17
Sub377	2016.11	master	17	2016.07	5 years（2011.09-2016.07）	17
Sub378	2016.05	bachelor	14	2018.07	5 years（2013.09-2018.07）	16
Sub379	2016.06	bachelor	15	2018.07	5 years（2013.09-2018.07）	17
Sub380	2016.07	bachelor	15	2018.07	5 years（2013.09-2018.07）	17
Sub381	2016.07	master	18	2015.07	5 years（2010.09-2015.07）	17
Sub382	2016.07	bachelor	17	2016.07	5 years（2011.09-2016.07）	17
Sub383	2016.07	master	19	2014.07	5 years（2009.09-2014.07）	17
Sub384	2016.07	bachelor	16	2017.07	5 years（2012.09-2017.07）	17
Sub385	2016.07	bachelor	17	2017.07	5 years（2012.09-2017.07）	18
Sub386	2016.07	bachelor	17	2017.07	5 years（2012.09-2017.07）	18
Sub387	2016.07	bachelor	17	2017.07	5 years（2012.09-2017.07）	18
Sub388	2016.07	bachelor	16	2017.07	5 years（2012.09-2017.07）	17
Sub389	2016.07	bachelor	16	2017.07	5 years（2012.09-2017.07）	17

Sub390	2016.07	master	19	2013.07	5 years（2008.09-2013.07）	16
Sub391	2016.08	bachelor	15	2017.07	5 years（2012.09-2017.07）	16
Sub392	2016.08	master	17	2016.07	5 years（2011.09-2016.07）	17
Sub393	2016.08	master	17	2015.07	5 years（2010.09-2015.07）	16
Sub394	2016.09	bachelor	17	2019.07	5 years（2014.09-2019.07）	20
Sub395	2016.09	bachelor	16	2017.07	5 years（2012.09-2017.07）	17
Sub396	2016.09	bachelor	17	2017.07	5 years（2012.09-2017.07）	18
Sub397	2016.09	master	17	2016.07	5 years（2011.09-2016.07）	17
Sub398	2016.09	bachelor	15	2018.07	5 years（2013.09-2018.07）	17
Sub399	2016.09	bachelor	16	2018.07	5 years（2013.09-2018.07）	18
Sub400	2016.09	bachelor	14	2019.07	5 years（2014.09-2019.07）	17
Sub401	2016.09	bachelor	16	2017.07	5 years（2012.09-2017.07）	17
Sub402	2016.09	bachelor	14	2019.07	5 years（2014.09-2019.07）	17
Sub403	2016.09	bachelor	15	2019.07	5 years（2014.09-2019.07）	18
Sub404	2016.10	bachelor	13	2020.07	5 years（2015.09-2020.07）	17
Sub405	2016.10	bachelor	13	2020.07	5 years（2015.09-2020.07）	17
Sub406	2016.10	bachelor	13	2020.07	5 years（2015.09-2020.07）	17
Sub407	2016.10	master	16	2015.07	5 years（2010.09-2015.07）	15
Sub408	2016.11	master	20	2016.07	5 years（2011.09-2016.07）	20
Sub409	2016.11	master	17	2016.07	5 years（2011.09-2016.07）	17
Sub410	2016.11	bachelor	17	2017.07	5 years（2012.09-2017.07）	18
Sub411	2016.11	bachelor	17	2018.07	5 years（2013.09-2018.07）	19
Sub412	2016.12	master	18	2016.07	5 years（2011.09-2016.07）	18
Sub413	2016.12	bachelor	12	2020.07	4 years（2016.09-2020.07）	16
Sub414	2016.12	master	17	2016.07	5 years（2011.09-2016.07）	17
Sub415	2016.12	bachelor	14	2020.07	5 years（2015.09-2020.07）	18
Sub416	2016.12	bachelor	16	2017.07	5 years（2012.09-2017.07）	17
Sub417	2016.12	bachelor	16	2017.07	5 years（2012.09-2017.07）	17
Sub418	2016.12	master	17	2016.07	5 years（2011.09-2016.07）	17
Sub419	2016.04	bachelor	15	2018.07	5 years（2013.09-2018.07）	17
Sub420	2016.04	bachelor	15	2018.07	5 years（2013.09-2018.07）	17
Sub421	2016.04	bachelor	14	2018.07	5 years（2013.09-2018.07）	16
Sub422	2016.04	bachelor	15	2018.07	5 years（2013.09-2018.07）	17
Sub423	2016.03	master	19	2014.07	5 years（2009.09-2014.07）	17
Sub424	2016.04	bachelor	17	2016.07	5 years（2011.09-2016.07）	17
Sub425	2016.05	bachelor	15	2018.07	5 years（2013.09-2018.07）	17
Sub426	2016.04	bachelor	15	2018.07	5 years（2013.09-2018.07）	17
Sub427	2016.05	bachelor	13	2019.07	5 years（2014.09-2019.07）	16
Sub428	2016.05	bachelor	14	2019.07	5 years（2014.09-2019.07）	17
Sub429	2016.05	bachelor	16	2016.07	5 years（2011.09-2016.07）	16
Sub430	2016.04	bachelor	16	2016.07	5 years（2011.09-2016.07）	16
Sub431	2016.04	bachelor	17	2016.07	5 years（2011.09-2016.07）	17
Sub432	2016.04	bachelor	18	2016.07	5 years（2011.09-2016.07）	18
Sub433	2016.05	bachelor	17	2016.07	5 years（2011.09-2016.07）	17

Sub434	2016.05	bachelor	17	2016.07	5 years（2011.09-2016.07）	17
Sub435	2016.06	bachelor	15	2018.07	5 years（2013.09-2018.07）	17
Sub436	2016.06	bachelor	15	2018.07	5 years（2013.09-2018.07）	17
Sub437	2016.06	bachelor	14	2018.07	5 years（2013.09-2018.07）	16
Sub438	2016.06	bachelor	15	2018.07	5 years（2013.09-2018.07）	17
Sub439	2016.06	bachelor	17	2016.07	5 years（2011.09-2016.07）	17
Sub440	2016.07	bachelor	17	2016.07	5 years（2011.09-2016.07）	17
Sub441	2016.07	bachelor	17	2016.07	5 years（2011.09-2016.07）	17
Sub442	2016.10	bachelor	14	2019.07	5 years（2014.09-2019.07）	17
Sub443	2016.06	bachelor	15	2018.07	5 years（2013.09-2018.07）	17
Sub444	2016.06	bachelor	16	2018.07	5 years（2013.09-2018.07）	18
Sub445	2016.11	bachelor	15	2018.07	5 years（2013.09-2018.07）	17
Sub446	2016.10	master	18	2013.07	5 years（2008.09-2013.07）	15
Sub447	2017.04	bachelor	16	2017.07	5 years（2012.09-2017.07）	16
Sub448	2017.04	bachelor	16	2017.07	5 years（2012.09-2017.07）	16

Supplementary Table 3. Courses classifications and the number of participants

Classifications	Courses	Participants
Scientific Method		
	Advanced Mathematics	448
	Basic Chemistry	337
	Computer Basics	422
	Linear Algebra and Probability Theory	336
	Organic Chemistry	447
	Physics	446
Behavioural and Social Sciences		
	Behavioral Medicine	273
	Doctor-Patient Communication	275
	Early Patient Contact Clinical Practice Training	331
	Epidemiology	324
	History of Medicine and Introduction to Medicine	323
	Ideological and Moral Cultivation and Legal Basis	448
	Medical Ethics	431
	Medical Psychology	341
	Medical Statistics	448
	Preventive Medicine	307
	Social Medicine	276
Basic Biomedical Sciences		
	Biochemistry	448
	Cell Biology	446
	Functional Experiment	324
	Genetics	322
	Histology and Embryology	447
	Immunology	448
	Integrated Curriculum of Basic Medicine	249
	Microbiology	448

	Molecular Biology	142
	Parasitology	447
	Pathology	369
	Pathophysiology	448
	Pharmacology	448
	Physiology	448
	Regional Anatomy	344
	Sectional Anatomy	234
	Systematic Anatomy	448
Clinical Sciences and Skills		
	Clinical Skills Science	291
	Dermatovenereology	307
	Diagnostics	448
	Emergency Medicine	276
	Evidence-Based Medicine	256
	Forensic Medicine	184
	General Practice	256
	Geriatrics	304
	Gynaecology and Obstetrics	423
	Infectious Diseases	276
	Integrated Curriculum of Clinical Medicine	379
	Internal Medicine	448
	Medical Imaging	440
	Neurology	422
	Oncology and Radiotherapy	298
	Operative Surgery	447
	Ophthalmology	422
	Otorhinolaryngology	423
	Pediatrics	448
	Psychiatry	307

	Rehabilitation Medicine	178
	Stomatology	376
	Surgery (with Anesthesiology)	448
	Traditional Chinese Medicine	307

Supplementary Table 4. The Academic Grading System of Tianjin Medical University

Scale (Hundred-mark system)	US Grade	Converted Points
90.0 - 100.0	A	4.0
85.0 - 89.9	A-	3.7
82.0 - 84.9	B+	3.3
78.0 - 81.9	B	3.0
75.0 - 77.9	B-	2.7
72.0 - 74.9	C+	2.3
69.0 - 71.9	C	2.0
65.0 - 68.9	C-	1.7
61.0 - 64.9	D+	1.3
60.0 - 60.9	D	1.0
0.0 - 59.9	F	0.0

Supplementary Table 5. Personality items retained in the present study.

Scale	Items	Assessment content and scientific significance	Types of Variables
**Tridimensional Personality Questionnaire (TPQ)**	Novelty seeking	Novelty seeking is referred to the exploratory excitability in response to novel stimuli, which is also characterized by extravagance, disorderliness, and impulsivity.	Continuous Variables
	Harm avoidance	Harm avoidance is referred to behavioral inhibition in response to dangerous or abhorrent stimuli.	Continuous Variables
	Reward dependence	Reward dependence is referred to the tendency to respond particularly to signals of social reward.	Continuous Variables
**Neuroticism Extraversion Openness (NEO) Personality Inventory**	Agreeableness	Agreeableness represents how you tend to behave in relationships with others, which is characterized by altruistic, warm, friendly, sympathetic and trustworthy.	Continuous Variables
	Conscientiousness	Conscientiousness denotes self-discipline, competence and achievement striving. A higher score represents well-organized, efficient and hard-working.	Continuous Variables
	Extraversion	Extraversion comprises outgoing, which is referred to how you interact with other people and the extent of seeking stimulation from others.	Continuous Variables
	Neuroticism	Neuroticism referred to the extent of sensitivity and nervous, which is characterized by anxiety, depression and proneness to experience negative affect	Continuous Variables
	Openness to Experience	Openness is referred to fantasy and aesthetics, a high openness score denotes creativity and invention.	Continuous Variables

Supplementary Table 6. The academic grading system based on theory of "point to point"

Converted grade point	Scale
0	score ≤ 59
(score/10)-5	60 ≤ score ≤ 89
4	90 ≤ score ≤ 100

Supplementary Table 7. The *rho* values derived from correlation analysis among academic achievements

	Scientific method	Behavioral and social sciences	Basic biomedical sciences	Clinical sciences and skills
Scientific method	-	0.51	0.70	0.55
Behavioral and social sciences	-	-	0.61	0.59
Basic biomedical sciences	-	-	-	0.76
Clinical sciences and skills	-	-	-	-

Supplementary Table 8. Correlations between TPQ, NEO and the total academic achievements

	P	*β*
**NS (TPQ)**	**9.71E-05**	**-3.93**
HA (TPQ)	6.52E-01	-0.45
RD(TPQ)	1.27E-01	1.53
Agreeableness (NEO)	2.11E-01	1.25
**Conscientiousness (NEO)**	**1.37E-04**	**3.86**
Extraversion (NEO)	2.82E-01	1.08
Neuroticism (NEO)	5.01E-01	0.67
Openness (NEO)	1.40E-01	-1.48

Notes: The β and P values were obtained from the linear regression estimate, and the bold represented the significant correlations. Abbreviations: HA = Harm Avoidance; NS = Novelty Seeking; RD = Reward Dependence.

Supplementary Table 9. Correlations between TPQ and academic achievements after time gap regression

	Scientific Method		Behavioural and Social Sciences		Basic Biomedical Sciences		Clinical Sciences and Skills	
	P	*β*	*P*	*β*	*P*	*β*	*P*	*β*
**NS**	**4.58E-04**	**-3.53**	**1.43E-05**	**-4.39**	**3.56E-04**	**-3.60**	**9.53E-03**	**-2.60**
HA	6.34E-01	-0.48	2.07E-01	-1.26	9.54E-01	0.06	8.20E-01	-0.23
RD	1.21E-01	1.55	4.25E-01	0.80	2.31E-01	1.20	1.59E-01	1.41

Notes: The β and P values were obtained from the linear regression estimate, and the bold represented the significant correlations. Abbreviations: HA = Harm Avoidance; NS = Novelty Seeking; RD = Reward Dependence.

Supplementary Table 10. Correlations between the NEO personality inventory and academic achievements after time gap regression

	Scientific Method		Behavioural and Social Sciences		Basic Biomedical Sciences		Clinical Sciences and Skills	
	P	*β*	*P*	*β*	*P*	*β*	*P*	*β*
Agreeableness	1.80E-01	1.34	6.42E-01	-0.47	2.20E-01	1.23	2.15E-01	1.24
**Conscientiousness**	**1.55E-04**	**3.82**	**5.19E-04**	**3.50**	**3.37E-03**	**2.95**	**8.43E-03**	**2.65**
Extraversion	4.46E-01	0.76	3.70E-01	0.90	5.14E-01	0.65	1.84E-01	1.33
Neuroticism	4.41E-01	0.77	6.53E-01	0.45	9.02E-01	0.12	3.33E-01	0.97
Openness	1.32E-01	-1.51	8.54E-01	-0.18	1.96E-01	-1.29	1.29E-01	-1.52

Notes: The β and P values were obtained from the linear regression estimate, and the bold represented the significant correlations.

Supplementary Table 11. The 95% confidence intervals of indirect effects mediated by brain functional property based on Automatic Anatomical Labeling (AAL) mask

Brain area (AAL)	Conscientiousness				Novelty seeking			
	ReHo	FCD	FCS	ALFF	ReHo	FCD	FCS	ALFF
Precentral_L	(-0.0006,0.0038)	(-0.0009,0.0035)	(-0.0017,0.0018)	(-0.0014,0.0015)	(-0.0035,0.0009)	(-0.0026,0.0019)	(-0.0013,0.0022)	(-0.0013,0.0013)
Precentral_R	(-0.0007,0.0038)	(-0.0006,0.0037)	(-0.0013,0.003)	(-0.0008,0.0021)	(-0.004,0.0005)	(-0.0033,0.0012)	(-0.0019,0.0023)	(-0.0023,0.0006)
Frontal_Sup_L	(-0.004,0.0013)	(-0.0005,0.0005)	(-0.0016,0.0011)	(-0.0013,0.0026)	(-0.0041,0.0012)	(-0.002,0.0007)	(-0.0009,0.0022)	(-0.0036,0.0005)
Frontal_Sup_R	(-0.0033,0.0017)	(-0.0003,0.0003)	(-0.0016,0.0014)	(-0.001,0.0016)	(-0.0066,-0.0002)	(-0.0021,0.0011)	(-0.0022,0.0008)	(-0.0036,0.0005)
Frontal_Sup_Orb_L	(-0.0019,0.0009)	(-0.0007,0.0012)	(-0.0013,0.0005)	(-0.0009,0.0005)	(-0.003,0.0006)	(-0.0005,0.0008)	(-0.0005,0.0003)	(-0.0005,0.0009)
Frontal_Sup_Orb_R	(-0.002,0.0013)	(-0.0006,0.0008)	(-0.0008,0.0007)	(-0.0014,0.0006)	(-0.0042,0.0003)	(-0.0006,0.001)	(-0.0011,0.0008)	(-0.0014,0.0007)
Frontal_Mid_L	(-0.003,0.0016)	(-0.0003,0.0003)	(-0.0003,0.0002)	(-0.0009,0.0019)	(-0.0047,0.0004)	(-0.0018,0.0007)	(-0.0005,0.0009)	(-0.0049,0.0001)
Frontal_Mid_R	(-0.0036,0.0006)	(-0.0002,0.0002)	(-0.0005,0.0007)	(-0.0009,0.0007)	(-0.0046,0.0002)	(-0.0024,0.0013)	(-0.0015,0.0013)	(-0.0031,0.0008)
Frontal_Mid_Orb_L	(-0.0028,0.0011)	(-0.001,0.001)	(-0.0003,0.0003)	(-0.0021,0.0008)	(-0.0038,0.0006)	(-0.0003,0.0004)	(-0.0004,0.0006)	(-0.0027,0.001)
Frontal_Mid_Orb_R	(-0.0009,0.0017)	(-0.0007,0.0006)	(-0.0013,0.0006)	(-0.0005,0.0012)	(-0.0017,0.0009)	(-0.0006,0.0006)	(-0.0004,0.0006)	(-0.0009,0.0013)
Frontal_Inf_Oper_L	(-0.0008,0.0005)	(-0.0007,0.001)	(-0.0007,0.0015)	(-0.0006,0.0004)	(-0.001,0.0007)	(-0.0007,0.0021)	(-0.0011,0.0015)	(-0.0019,0.0007)
Frontal_Inf_Oper_R	(-0.0003,0.0002)	(-0.0004,0.0004)	(-0.0009,0.0005)	(-0.0017,0.001)	(-0.0007,0.0017)	(-0.0007,0.0019)	(-0.0006,0.0015)	(-0.0031,0.0005)
Frontal_Inf_Tri_L	(-0.0021,0.0011)	(-0.0005,0.0003)	(-0.0008,0.002)	(-0.0008,0.0011)	(-0.0036,0.0004)	(-0.001,0.0016)	(-0.0016,0.0022)	(-0.0036,0.0006)
Frontal_Inf_Tri_R	(-0.0028,0.001)	(-0.0003,0.0003)	(-0.0025,0.0007)	(-0.0017,0.0009)	(-0.0021,0.0006)	(-0.0012,0.0012)	(-0.0009,0.0022)	(-0.0027,0.0006)
Frontal_Inf_Orb_L	(-0.0006,0.0007)	(-0.0002,0.0002)	(-0.0006,0.0008)	(-0.0005,0.0004)	(-0.0018,0.0009)	(-0.0008,0.0012)	(-0.0033,0.0008)	(-0.0008,0.0006)
Frontal_Inf_Orb_R	(-0.001,0.0005)	(-0.0006,0.0005)	(-0.0014,0.0005)	(-0.0006,0.0007)	(-0.0004,0.0004)	(-0.0005,0.0009)	(-0.0008,0.0008)	(-0.0007,0.0007)
Rolandic_Oper_L	(-0.0006,0.0005)	(-0.0005,0.0004)	(-0.001,0.0005)	(-0.0003,0.0003)	(-0.0006,0.0004)	(-0.0006,0.0004)	(-0.0006,0.0015)	(-0.0008,0.0014)
Rolandic_Oper_R	(-0.0005,0.0006)	(-0.0005,0.0008)	(-0.0012,0.0005)	(-0.0013,0.0014)	(-0.0001,0.0001)	(-0.0007,0.0005)	(-0.0007,0.0018)	(-0.0015,0.0022)

Supp_Motor_Area_L	(-0.0014,0.0012)	(-0.0005,0.0004)	(-0.0006,0.0005)	(-0.0024,0.0011)	(-0.0005,0.0031)	(-0.0005,0.0004)	(-0.0006,0.0005)	(-0.0018,0.0022)
Supp_Motor_Area_R	(-0.0005,0.0005)	(-0.0003,0.0004)	(-0.0004,0.0004)	(-0.0019,0.0005)	(-0.0005,0.0007)	(-0.0004,0.0006)	(-0.0001,0.0001)	(-0.0007,0.0006)
Olfactory_L	(-0.0014,0.0006)	(-0.0004,0.0004)	(-0.0008,0.0007)	(-0.0014,0.0019)	(-0.0008,0.0005)	(-0.0011,0.0013)	(-0.00004,0.00004)	(-0.0021,0.0012)
Olfactory_R	(-0.0014,0.001)	(-0.0005,0.0004)	(-0.001,0.0007)	(-0.0014,0.0013)	(-0.0017,0.0009)	(-0.001,0.001)	(-0.0014,0.0006)	(-0.0013,0.0009)
Frontal_Sup_Medial_L	(-0.0026,0.0019)	(-0.0002,0.0002)	(-0.0027,0.0008)	(-0.0016,0.0017)	(-0.0043,0.0005)	(-0.0017,0.0011)	(-0.0009,0.0024)	(-0.0039,0.0003)
Frontal_Sup_Medial_R	(-0.0015,0.002)	(-0.0004,0.0003)	(-0.0021,0.0014)	(-0.0011,0.0011)	(-0.0037,0.0006)	(-0.0011,0.0015)	(-0.0014,0.0023)	(-0.0021,0.0006)
Frontal_Mid_Orb_L	(-0.0024,0.003)	(-0.0006,0.0012)	(-0.0028,0.001)	(-0.001,0.0006)	(-0.0054,0.0006)	(-0.002,0.0008)	(-0.0013,0.0017)	(-0.0011,0.0007)
Frontal_Mid_Orb_R	(-0.002,0.0026)	(-0.0005,0.001)	(-0.0026,0.0011)	(-0.0019,0.0009)	(-0.0036,0.0012)	(-0.0015,0.001)	(-0.0009,0.0026)	(-0.0022,0.0006)
Rectus_L	(-0.0016,0.0014)	(-0.0007,0.0006)	(-0.0016,0.0009)	(-0.0014,0.0011)	(-0.0032,0.0005)	(-0.0017,0.0014)	(-0.0014,0.0011)	(-0.0017,0.0007)
Rectus_R	(-0.0017,0.0016)	(-0.0008,0.0005)	(-0.0013,0.0008)	(-0.002,0.0016)	(-0.0034,0.0005)	(-0.0017,0.0013)	(-0.0013,0.001)	(-0.0045,0.0002)
Insula_L	(-0.0006,0.0007)	(-0.0007,0.0009)	(-0.001,0.0005)	(-0.0013,0.0013)	(-0.0013,0.0007)	(-0.001,0.0006)	(-0.001,0.001)	(-0.0015,0.002)
Insula_R	(-0.0004,0.0008)	(-0.0007,0.0009)	(-0.0012,0.0014)	(-0.0017,0.0017)	(-0.0003,0.0003)	(-0.0007,0.0005)	(-0.0006,0.0015)	(-0.0017,0.0013)
Cingulum_Ant_L	(-0.0015,0.0008)	(-0.00002,0.00002)	(-0.0017,0.0005)	(-0.0032,0.0016)	(-0.0007,0.0007)	(-0.0006,0.0009)	(-0.0005,0.0008)	(-0.005,0.0005)
Cingulum_Ant_R	(-0.0009,0.0008)	(-0.0003,0.0002)	(-0.0013,0.0006)	(-0.0028,0.0021)	(-0.0004,0.0005)	(-0.0007,0.0015)	(-0.0007,0.0007)	(-0.0075,-0.0009)
Cingulum_Mid_L	(-0.0011,0.0009)	(-0.0005,0.0004)	(-0.0005,0.0005)	(-0.0007,0.0018)	(-0.0007,0.0005)	(-0.0007,0.0005)	(-0.0003,0.0005)	(-0.0026,0.0008)
Cingulum_Mid_R	(-0.0004,0.0006)	(-0.0005,0.0003)	(-0.0006,0.0004)	(-0.0014,0.001)	(-0.0003,0.0003)	(-0.0007,0.0007)	(-0.0008,0.0011)	(-0.0026,0.0008)
Cingulum_Post_L	(-0.0011,0.0021)	(-0.0005,0.0013)	(-0.0017,0.0009)	(-0.001,0.0011)	(-0.0032,0.0006)	(-0.0027,0.0009)	(-0.0016,0.001)	(-0.0021,0.0007)
Cingulum_Post_R	(-0.0014,0.0017)	(-0.0007,0.0013)	(-0.0015,0.0011)	(-0.0008,0.0022)	(-0.0028,0.0007)	(-0.0024,0.0009)	(-0.0022,0.0009)	(-0.0028,0.0011)
Hippocampus_L	(-0.0006,0.0004)	(-0.0011,0.0006)	(-0.001,0.0014)	(-0.0005,0.0007)	(-0.0006,0.0005)	(-0.0009,0.0011)	(-0.0002,0.0002)	(-0.003,0.0007)
Hippocampus_R	(-0.0008,0.0005)	(-0.0018,0.0006)	(-0.0007,0.001)	(-0.0005,0.0006)	(-0.0004,0.0005)	(-0.0013,0.0009)	(-0.0008,0.0005)	(-0.0013,0.0013)
ParaHippocampal_L	(-0.0001,0.0001)	(-0.0004,0.0003)	(-0.0006,0.0017)	(-0.0007,0.0009)	(-0.0011,0.0006)	(-0.0003,0.0003)	(-0.0011,0.0006)	(-0.0006,0.0013)
ParaHippocampal_R	(-0.0003,0.0002)	(-0.0007,0.0004)	(-0.0005,0.0012)	(-0.0012,0.002)	(-0.0001,0.0001)	(-0.0004,0.0004)	(-0.0011,0.0007)	(-0.0006,0.0031)
Amygdala_L	(-0.0002,0.0003)	(-0.0003,0.0003)	(-0.0005,0.0013)	(-0.0003,0.0003)	(-0.0007,0.001)	(-0.0004,0.0003)	(-0.0006,0.0011)	(-0.0018,0.0011)
Amygdala_R	(-0.0001,0.0001)	(-0.0004,0.0005)	(-0.0005,0.0005)	(-0.0001,0.0001)	(-0.0006,0.0005)	(-0.0002,0.0002)	(-0.0012,0.0011)	(-0.0011,0.0005)
Calcarine_L	(-0.0005,0.0016)	(-0.0005,0.0023)	(-0.0003,0.0004)	(-0.0012,0.0032)	(-0.0012,0.0011)	(-0.0014,0.0006)	(-0.0005,0.0004)	(-0.0036,0.0004)
Calcarine_R	(-0.0006,0.0013)	(-0.0008,0.0017)	(-0.0005,0.0005)	(-0.0017,0.0021)	(-0.0011,0.0009)	(-0.0015,0.0006)	(-0.0005,0.0005)	(-0.0031,0.0006)

Cuneus_L	(-0.0004,0.0008)	(-0.0009,0.0013)	(-0.001,0.0007)	(-0.002,0.0029)	(-0.0014,0.0007)	(-0.0013,0.0006)	(-0.0005,0.0008)	(-0.0056,0.0002)
Cuneus_R	(-0.0006,0.0007)	(-0.0012,0.0015)	(-0.0004,0.0003)	(-0.0026,0.0031)	(-0.001,0.0006)	(-0.0016,0.0007)	(-0.0003,0.0004)	(-0.0057,0.0005)
Lingual_L	(-0.0015,0.0017)	(-0.0007,0.002)	(-0.0001,0.0001)	(-0.002,0.0028)	(-0.0024,0.0006)	(-0.0012,0.0006)	(-0.0012,0.0007)	(-0.0052,0.00005)
Lingual_R	(-0.002,0.0012)	(-0.0012,0.0013)	(-0.0003,0.0002)	(-0.0016,0.0025)	(-0.0021,0.0007)	(-0.001,0.0006)	(-0.0008,0.0006)	(-0.0034,0.0005)
Occipital_Sup_L	(-0.0008,0.0005)	(-0.0005,0.0009)	(-0.0012,0.0006)	(-0.002,0.0024)	(-0.0004,0.0004)	(-0.0003,0.0003)	(-0.0002,0.0001)	(-0.0036,0.0012)
Occipital_Sup_R	(-0.0009,0.0005)	(-0.001,0.0007)	(-0.0006,0.0004)	(-0.0025,0.0014)	(-0.0005,0.0009)	(-0.0005,0.0004)	(-0.0001,0.0001)	(-0.0026,0.0017)
Occipital_Mid_L	(-0.0018,0.0006)	(-0.0008,0.0006)	(-0.0008,0.0005)	(-0.0026,0.0019)	(-0.0006,0.001)	(-0.0006,0.0005)	(-0.0004,0.0004)	(-0.003,0.0016)
Occipital_Mid_R	(-0.0007,0.0013)	(-0.001,0.0005)	(-0.0011,0.0006)	(-0.0024,0.0006)	(-0.0007,0.0031)	(-0.0003,0.0002)	(-0.0003,0.0003)	(-0.001,0.0007)
Occipital_Inf_L	(-0.001,0.0006)	(-0.0012,0.0011)	(-0.0012,0.0005)	(-0.0028,0.0021)	(-0.0004,0.0006)	(-0.0005,0.0006)	(-0.0012,0.0013)	(-0.0033,0.0017)
Occipital_Inf_R	(-0.0016,0.0006)	(-0.0012,0.0007)	(-0.0003,0.0003)	(-0.0027,0.0012)	(-0.0008,0.0009)	(-0.0002,0.0002)	(-0.0008,0.0016)	(-0.0017,0.0011)
Fusiform_L	(-0.0012,0.0016)	(-0.0012,0.0009)	(-0.0005,0.0004)	(-0.0015,0.0019)	(-0.0022,0.0008)	(-0.0005,0.0007)	(-0.0008,0.0007)	(-0.0049,0.00002)
Fusiform_R	(-0.0015,0.0018)	(-0.0018,0.0009)	(-0.0017,0.0008)	(-0.0011,0.0008)	(-0.0021,0.001)	(-0.0008,0.0009)	(-0.0021,0.0006)	(-0.0023,0.0007)
Postcentral_L	(-0.0012,0.0031)	(-0.0007,0.0028)	(-0.0013,0.0015)	(-0.0014,0.0022)	(-0.0045,0.0002)	(-0.0028,0.0008)	(-0.0011,0.0017)	(-0.0041,0.0003)
Postcentral_R	(-0.0009,0.0034)	(-0.0008,0.0025)	(-0.0015,0.0021)	(-0.0008,0.0016)	(-0.0045,0.0004)	(-0.0026,0.0009)	(-0.002,0.0017)	(-0.0023,0.0006)
Parietal_Sup_L	(-0.0015,0.0009)	(-0.0009,0.0006)	(-0.0012,0.0012)	(-0.0026,0.0013)	(-0.0027,0.0006)	(-0.0006,0.0015)	(-0.002,0.0008)	(-0.0028,0.0019)
Parietal_Sup_R	(-0.001,0.0007)	(-0.0013,0.0008)	(-0.0012,0.001)	(-0.0018,0.0012)	(-0.0019,0.0007)	(-0.0006,0.0021)	(-0.0011,0.0012)	(-0.0008,0.0022)
Parietal_Inf_L	(-0.0002,0.0002)	(-0.0002,0.0002)	(-0.0014,0.0012)	(-0.0002,0.0002)	(-0.0014,0.0009)	(-0.0007,0.0006)	(-0.0014,0.0017)	(-0.0006,0.0008)
Parietal_Inf_R	(-0.0003,0.0004)	(-0.0007,0.0008)	(-0.0011,0.0006)	(-0.0007,0.0006)	(-0.0005,0.0007)	(-0.0009,0.0019)	(-0.0009,0.0017)	(-0.0019,0.0007)
SupraMarginal_L	(-0.0002,0.0002)	(-0.0003,0.0004)	(-0.0005,0.0005)	(-0.0005,0.0003)	(-0.0005,0.0006)	(-0.0016,0.001)	(-0.0006,0.0017)	(-0.0008,0.0005)
SupraMarginal_R	(-0.0001,0.0002)	(-0.0003,0.0005)	(-0.001,0.0006)	(-0.0011,0.001)	(-0.0006,0.001)	(-0.0016,0.0014)	(-0.0006,0.0027)	(-0.0028,0.0006)
Angular_L	(-0.0012,0.0012)	(-0.0003,0.0004)	(-0.0013,0.0012)	(-0.0005,0.0004)	(-0.0016,0.0011)	(-0.0014,0.0006)	(-0.0009,0.0011)	(-0.0004,0.0005)
Angular_R	(-0.0013,0.001)	(-0.0004,0.0006)	(-0.001,0.0009)	(-0.0008,0.0014)	(-0.0009,0.0015)	(-0.002,0.0006)	(-0.0007,0.0008)	(-0.0026,0.0006)
Precuneus_L	(-0.0004,0.003)	(-0.0008,0.0008)	(-0.0005,0.001)	(-0.0014,0.0005)	(-0.0015,0.0012)	(-0.0016,0.0016)	(-0.0006,0.0007)	(-0.0008,0.0015)
Precuneus_R	(-0.0012,0.0027)	(-0.0007,0.0007)	(-0.0009,0.0013)	(-0.0008,0.0005)	(-0.002,0.0018)	(-0.0015,0.0012)	(-0.0016,0.001)	(-0.0006,0.0012)
Paracentral_Lobule_L	(-0.0007,0.0016)	(-0.0013,0.0017)	(-0.0015,0.0024)	(-0.0008,0.001)	(-0.0026,0.0006)	(-0.0032,0.0008)	(-0.0042,0.0004)	(-0.0016,0.001)
Paracentral_Lobule_R	(-0.0007,0.0019)	(-0.0011,0.0019)	(-0.0012,0.0024)	(-0.0008,0.0014)	(-0.0032,0.0005)	(-0.0031,0.0006)	(-0.004,0.0003)	(-0.0022,0.0006)

Caudate_L	(-0.0007,0.0018)	(-0.0008,0.0007)	(-0.0012,0.0005)	(-0.0006,0.0012)	(-0.0013,0.001)	(-0.001,0.0008)	(-0.0008,0.0005)	(-0.0013,0.0012)
Caudate_R	(-0.0012,0.0015)	(-0.0006,0.001)	(-0.001,0.0006)	(-0.0005,0.0009)	(-0.0015,0.0015)	(-0.0009,0.0011)	(-0.0016,0.0006)	(-0.0008,0.0007)
Putamen_L	(-0.0005,0.0008)	(-0.0014,0.0008)	(-0.0003,0.0004)	(-0.0005,0.0007)	(-0.0011,0.0006)	(-0.001,0.0012)	(-0.0005,0.0007)	(-0.0006,0.0016)
Putamen_R	(-0.0013,0.001)	(-0.0013,0.0007)	(-0.0002,0.0002)	(-0.0007,0.0004)	(-0.0018,0.0007)	(-0.001,0.0012)	(-0.0008,0.001)	(-0.0016,0.0006)
Pallidum_L	(-0.0004,0.0007)	(-0.0013,0.0007)	(-0.0012,0.0005)	(-0.0002,0.0002)	(-0.0002,0.0003)	(-0.0016,0.0009)	(-0.0005,0.0004)	(-0.0009,0.0014)
Pallidum_R	(-0.0017,0.0016)	(-0.0007,0.0006)	(-0.0007,0.0005)	(-0.0011,0.0006)	(-0.0016,0.0019)	(-0.0011,0.0006)	(-0.001,0.0006)	(-0.0017,0.0006)
Thalamus_L	(-0.0002,0.0002)	(-0.0008,0.001)	(-0.0008,0.0005)	(-0.0007,0.0009)	(-0.0005,0.001)	(-0.0012,0.0006)	(-0.0016,0.001)	(-0.0011,0.0006)
Thalamus_R	(-0.0002,0.0002)	(-0.001,0.0012)	(-0.0009,0.0005)	(-0.0011,0.0009)	(-0.0006,0.0009)	(-0.0016,0.0008)	(-0.0017,0.0012)	(-0.0024,0.0006)
Heschl_L	(-0.0007,0.0006)	(-0.0001,0.0001)	(-0.0011,0.0005)	(-0.0006,0.0004)	(-0.0005,0.0004)	(-0.0004,0.0003)	(-0.0006,0.0015)	(-0.0005,0.0005)
Heschl_R	(-0.002,0.0005)	(-0.001,0.0006)	(-0.0017,0.0007)	(-0.0006,0.0005)	(-0.0019,0.0009)	(-0.0002,0.0002)	(-0.0006,0.003)	(-0.0005,0.0007)
Temporal_Sup_L	(-0.0012,0.0009)	(-0.0004,0.0004)	(-0.0016,0.0006)	(-0.0005,0.0003)	(-0.0012,0.0012)	(-0.0007,0.0005)	(-0.0006,0.0018)	(-0.0006,0.0008)
Temporal_Sup_R	(-0.0008,0.0006)	(-0.0013,0.0006)	(-0.0027,0.0004)	(-0.0012,0.0009)	(-0.0015,0.0012)	(-0.0005,0.0009)	(-0.0008,0.0018)	(-0.0015,0.0009)
Temporal_Pole_Sup_L	(-0.0007,0.0004)	(-0.0005,0.001)	(-0.0004,0.0004)	(-0.0016,0.0015)	(-0.0004,0.0006)	(-0.0012,0.0007)	(-0.0025,0.0007)	(-0.0016,0.0013)
Temporal_Pole_Sup_R	(-0.0003,0.0004)	(-0.0005,0.0012)	(-0.0005,0.0016)	(-0.0017,0.0011)	(-0.0008,0.0005)	(-0.0011,0.0006)	(-0.0005,0.0004)	(-0.0026,0.0007)
Temporal_Mid_L	(-0.0006,0.0004)	(-0.0012,0.0005)	(-0.0005,0.0009)	(-0.0004,0.0003)	(-0.001,0.0007)	(-0.0008,0.0017)	(-0.0007,0.001)	(-0.0011,0.0006)
Temporal_Mid_R	(-0.0005,0.001)	(-0.0015,0.0005)	(-0.0005,0.0007)	(-0.0005,0.0011)	(-0.0013,0.0007)	(-0.0007,0.0016)	(-0.0009,0.0015)	(-0.0028,0.0006)
Temporal_Pole_Mid_L	(-0.0009,0.0005)	(-0.0004,0.0005)	(-0.0005,0.0004)	(-0.0005,0.0011)	(-0.0008,0.0005)	(-0.0009,0.0009)	(-0.001,0.0008)	(-0.0009,0.0011)
Temporal_Pole_Mid_R	(-0.0006,0.001)	(-0.0008,0.0009)	(-0.001,0.0013)	(-0.0006,0.0021)	(-0.0017,0.0007)	(-0.0006,0.0007)	(-0.001,0.0015)	(-0.0013,0.0013)
Temporal_Inf_L	(-0.0008,0.0005)	(-0.0003,0.0003)	(-0.0006,0.0015)	(-0.0003,0.0003)	(-0.0008,0.0007)	(-0.0008,0.0012)	(-0.0027,0.0006)	(-0.0004,0.0004)
Temporal_Inf_R	(-0.001,0.0006)	(-0.0006,0.0005)	(-0.0004,0.0003)	(-0.0006,0.0009)	(-0.0014,0.0006)	(-0.0008,0.0015)	(-0.001,0.0012)	(-0.0017,0.0006)

Abbreviations: AAL, Automatic Anatomical Labeling; ReHo, Regional Homogeneity; ALFF, Amplitude of Low Frequency Fluctuation; FCD, Functional Connectivity Density; FCS, Functional Connectivity Strength.

Supplementary Table 12. The 95% confidence intervals of indirect effects mediated by personality based on Automatic Anatomical Labeling (AAL) mask

Brain area (AAL)	Conscientiousness				Novelty seeking			
	ReHo	FCD	FCS	ALFF	ReHo	FCD	FCS	ALFF
Precentral_L	(-0.1235,0.022)	(-0.00025,0.00007)	(-0.0547,0.0519)	(-0.0804,0.0782)	(-0.1119,0.0358)	(-0.00006,0.0001)	(-0.0487,0.0146)	(-0.1674,0.0423)
Precentral_R	(-0.0944,0.0202)	(-0.00025,0.00005)	(-0.0666,0.0288)	(-0.0933,0.0344)	(-0.0726,0.0203)	(-0.00005,0.0001)	(-0.065,0.0188)	(-0.0426,0.0196)
Frontal_Sup_L	(-0.1024,0.0345)	(-0.0001,0.00011)	(-0.0621,0.0427)	(-0.0376,0.0742)	(-0.0254,0.0776)	(-0.00003,0.00016)	(-0.0178,0.0291)	(-0.0167,0.0362)
Frontal_Sup_R	(-0.1051,0.0528)	(-0.00008,0.00013)	(-0.0624,0.0562)	(-0.0416,0.0677)	(-0.0439,0.1054)	(-0.00004,0.0002)	(-0.0161,0.0539)	(-0.0151,0.0417)
Frontal_Sup_Orb_L	(-0.0529,0.0238)	(-0.00029,0.00005)	(-0.0696,0.0239)	(-0.0673,0.024)	(-0.0205,0.0705)	(-0.00003,0.00045)	(-0.0119,0.04)	(-0.0174,0.0974)
Frontal_Sup_Orb_R	(-0.0464,0.0298)	(-0.00025,0.00007)	(-0.0465,0.0421)	(-0.0652,0.0221)	(-0.02,0.063)	(-0.00004,0.00037)	(-0.0128,0.0556)	(-0.0187,0.0833)
Frontal_Mid_L	(-0.087,0.0467)	(-0.00011,0.00011)	(-0.0443,0.0626)	(-0.0366,0.0771)	(-0.0266,0.0844)	(-0.00003,0.00014)	(-0.0152,0.0478)	(-0.0175,0.0349)
Frontal_Mid_R	(-0.131,0.0207)	(-0.00011,0.00011)	(-0.0275,0.078)	(-0.0563,0.0441)	(-0.0191,0.0684)	(-0.00003,0.00015)	(-0.0141,0.0466)	(-0.0135,0.0419)
Frontal_Mid_Orb_L	(-0.0514,0.0199)	(-0.00027,0.00003)	(-0.0422,0.0354)	(-0.0475,0.0172)	(-0.0157,0.0514)	(-0.00004,0.00038)	(-0.0146,0.0201)	(-0.0113,0.0419)
Frontal_Mid_Orb_R	(-0.0234,0.043)	(-0.00011,0.00013)	(-0.012,0.0789)	(-0.0142,0.039)	(-0.0149,0.0609)	(-0.00002,0.00038)	(-0.015,0.0198)	(-0.0098,0.0444)
Frontal_Inf_Oper_L	(-0.0775,0.0424)	(-0.00014,0.0001)	(-0.0602,0.0271)	(-0.0706,0.0414)	(-0.0614,0.0153)	(-0.00004,0.00009)	(-0.0416,0.0118)	(-0.0313,0.02)
Frontal_Inf_Oper_R	(-0.0658,0.055)	(-0.00011,0.00012)	(-0.0249,0.0713)	(-0.0663,0.0412)	(-0.0277,0.0243)	(-0.00003,0.00018)	(-0.0391,0.0123)	(-0.0159,0.0316)
Frontal_Inf_Tri_L	(-0.0791,0.0433)	(-0.00009,0.00014)	(-0.0647,0.0267)	(-0.0476,0.0659)	(-0.0229,0.028)	(-0.00003,0.00013)	(-0.0377,0.0124)	(-0.0158,0.0497)
Frontal_Inf_Tri_R	(-0.0939,0.035)	(-0.0001,0.00017)	(-0.0251,0.0915)	(-0.0782,0.0431)	(-0.0363,0.0213)	(-0.00004,0.00017)	(-0.0393,0.0144)	(-0.0161,0.0567)
Frontal_Inf_Orb_L	(-0.0431,0.0499)	(-0.00016,0.00015)	(-0.0577,0.0377)	(-0.0614,0.0563)	(-0.0129,0.0438)	(-0.00004,0.00039)	(-0.0258,0.0164)	(-0.0194,0.0993)
Frontal_Inf_Orb_R	(-0.0673,0.0318)	(-0.00014,0.00014)	(-0.0208,0.0907)	(-0.0518,0.0574)	(-0.0156,0.0319)	(-0.00003,0.00042)	(-0.0224,0.0218)	(-0.0218,0.1153)
Rolandic_Oper_L	(-0.0295,0.078)	(-0.00006,0.00013)	(-0.0257,0.0498)	(-0.0674,0.0486)	(-0.1308,0.0122)	(-0.00003,0.00006)	(-0.0338,0.0097)	(-0.0526,0.0158)
Rolandic_Oper_R	(-0.0279,0.0725)	(-0.00005,0.00014)	(-0.0178,0.0573)	(-0.0508,0.0543)	(-0.0738,0.0128)	(-0.00003,0.00007)	(-0.0395,0.0095)	(-0.0246,0.0226)

Supp_Motor_Area_L	(-0.0617,0.0547)	(-0.00023,0.0001)	(-0.0799,0.0262)	(-0.0852,0.0369)	(-0.0333,0.0215)	(-0.00004,0.00013)	(-0.0173,0.0293)	(-0.0368,0.0199)
Supp_Motor_Area_R	(-0.06,0.0592)	(-0.00014,0.00012)	(-0.0706,0.0338)	(-0.1398,0.0161)	(-0.0349,0.021)	(-0.00004,0.00011)	(-0.0155,0.0357)	(-0.0876,0.0158)
Olfactory_L	(-0.0781,0.016)	(-0.00015,0.00015)	(-0.0646,0.0139)	(-0.0385,0.0509)	(-0.0139,0.0809)	(-0.00004,0.00025)	(-0.011,0.0266)	(-0.0251,0.0864)
Olfactory_R	(-0.0508,0.0362)	(-0.00019,0.00013)	(-0.0431,0.0291)	(-0.0507,0.0486)	(-0.0237,0.0908)	(-0.00004,0.00021)	(-0.01,0.033)	(-0.0184,0.0822)
Frontal_Sup_Medial_L	(-0.0594,0.0445)	(-0.00007,0.00007)	(-0.0588,0.017)	(-0.042,0.0432)	(-0.0295,0.0845)	(-0.00002,0.00012)	(-0.0097,0.0245)	(-0.0183,0.0659)
Frontal_Sup_Medial_R	(-0.0428,0.0547)	(-0.00005,0.00008)	(-0.0433,0.0288)	(-0.0425,0.0405)	(-0.0295,0.0869)	(-0.00002,0.00012)	(-0.0099,0.0234)	(-0.0158,0.0723)
Frontal_Mid_Orb_L	(-0.0244,0.0301)	(-0.00004,0.00009)	(-0.0437,0.0155)	(-0.0268,0.0149)	(-0.0298,0.0508)	(-0.00003,0.00014)	(-0.0075,0.0199)	(-0.0054,0.0212)
Frontal_Mid_Orb_R	(-0.0257,0.0336)	(-0.00004,0.00011)	(-0.0458,0.0198)	(-0.039,0.019)	(-0.0245,0.057)	(-0.00003,0.00016)	(-0.0097,0.0181)	(-0.0096,0.0423)
Rectus_L	(-0.0364,0.0309)	(-0.00016,0.00013)	(-0.0501,0.0264)	(-0.0354,0.0288)	(-0.0242,0.0785)	(-0.00005,0.00037)	(-0.0099,0.0333)	(-0.0167,0.0714)
Rectus_R	(-0.0373,0.0344)	(-0.00018,0.00011)	(-0.0467,0.029)	(-0.0467,0.0375)	(-0.0249,0.0737)	(-0.00005,0.00033)	(-0.0103,0.0396)	(-0.0353,0.0899)
Insula_L	(-0.03,0.1046)	(-0.00004,0.00019)	(-0.0231,0.0652)	(-0.0601,0.0577)	(-0.1248,0.0141)	(-0.00003,0.00008)	(-0.0416,0.011)	(-0.0157,0.0506)
Insula_R	(-0.0388,0.0792)	(-0.00003,0.00016)	(-0.0033,0.0974)	(-0.0455,0.0454)	(-0.0584,0.0156)	(-0.00002,0.0001)	(-0.0257,0.0118)	(-0.0135,0.0301)
Cingulum_Ant_L	(-0.0738,0.0374)	(-0.0001,0.00011)	(-0.0561,0.0128)	(-0.0575,0.0292)	(-0.015,0.0727)	(-0.00003,0.00017)	(-0.0084,0.0386)	(-0.0112,0.0357)
Cingulum_Ant_R	(-0.0651,0.0557)	(-0.0001,0.00011)	(-0.0475,0.0194)	(-0.0606,0.0444)	(-0.0158,0.0756)	(-0.00002,0.00017)	(-0.0092,0.0429)	(-0.0144,0.0331)
Cingulum_Mid_L	(-0.0171,0.1359)	(-0.00008,0.00016)	(-0.0651,0.024)	(-0.0381,0.1031)	(-0.171,0.0245)	(-0.00004,0.00008)	(-0.0108,0.0579)	(-0.2197,-0.0047)
Cingulum_Mid_R	(-0.0413,0.1016)	(-0.00008,0.00012)	(-0.0658,0.0254)	(-0.0968,0.0696)	(-0.1348,0.0174)	(-0.00003,0.0001)	(-0.0113,0.0678)	(-0.2244,0.0002)
Cingulum_Post_L	(-0.0285,0.0529)	(-0.00002,0.00008)	(-0.0398,0.0213)	(-0.0393,0.043)	(-0.0139,0.024)	(-0.00001,0.00006)	(-0.0085,0.0344)	(-0.0457,0.0107)
Cingulum_Post_R	(-0.0393,0.0459)	(-0.00002,0.00014)	(-0.0429,0.0328)	(-0.026,0.0688)	(-0.0269,0.0152)	(-0.00002,0.00009)	(-0.0108,0.0415)	(-0.0461,0.0132)
Hippocampus_L	(-0.0587,0.0907)	(-0.00019,0.00034)	(-0.1116,0.0073)	(-0.0972,0.0711)	(-0.1063,0.0213)	(-0.00005,0.00046)	(-0.0413,0.0141)	(-0.0482,0.0293)
Hippocampus_R	(-0.0391,0.099)	(-0.00009,0.00037)	(-0.0591,0.0401)	(-0.0533,0.1466)	(-0.1579,0.0233)	(-0.00005,0.00035)	(-0.0831,0.0154)	(-0.0259,0.098)
ParaHippocampal_L	(-0.0677,0.0625)	(-0.00014,0.00026)	(-0.0947,0.0126)	(-0.0397,0.0497)	(-0.0154,0.1008)	(-0.00005,0.00035)	(-0.0211,0.0219)	(-0.0124,0.0634)
ParaHippocampal_R	(-0.0731,0.0625)	(-0.0001,0.00023)	(-0.0717,0.0279)	(-0.038,0.0626)	(-0.0234,0.0416)	(-0.00004,0.00027)	(-0.056,0.0127)	(-0.0249,0.0978)
Amygdala_L	(-0.0484,0.0392)	(-0.00016,0.00019)	(-0.0499,0.0149)	(-0.0428,0.0224)	(-0.0138,0.0305)	(-0.00007,0.00009)	(-0.0138,0.0131)	(-0.0079,0.0464)
Amygdala_R	(-0.0454,0.0434)	(-0.00009,0.00021)	(-0.0495,0.0185)	(-0.0431,0.0327)	(-0.0117,0.0451)	(-0.00004,0.00025)	(-0.0129,0.0154)	(-0.0096,0.0479)
Calcarine_L	(-0.06,0.0113)	(-0.00011,0.00002)	(-0.0348,0.0304)	(-0.0288,0.0108)	(-0.0306,0.0089)	(-0.00002,0.00006)	(-0.0221,0.0103)	(-0.054,0.0241)
Calcarine_R	(-0.0401,0.0191)	(-0.00008,0.00004)	(-0.0497,0.0176)	(-0.0247,0.0197)	(-0.0372,0.0079)	(-0.00001,0.00006)	(-0.0315,0.0085)	(-0.0477,0.0151)

Cuneus_L	(-0.0512,0.0263)	(-0.00006,0.00004)	(-0.0125,0.062)	(-0.0253,0.0177)	(-0.0388,0.0105)	(-0.00002,0.00004)	(-0.0318,0.0089)	(-0.0512,0.0477)
Cuneus_R	(-0.0389,0.036)	(-0.00006,0.00005)	(-0.0261,0.039)	(-0.0264,0.0224)	(-0.0378,0.0103)	(-0.00002,0.00004)	(-0.0349,0.0083)	(-0.05,0.039)
Lingual_L	(-0.0326,0.0287)	(-0.00009,0.00003)	(-0.0346,0.0378)	(-0.031,0.0219)	(-0.0497,0.0113)	(-0.00002,0.00006)	(-0.0331,0.0099)	(-0.0642,0.0382)
Lingual_R	(-0.025,0.0426)	(-0.00006,0.00006)	(-0.0405,0.031)	(-0.0388,0.0249)	(-0.0613,0.0129)	(-0.00002,0.00005)	(-0.0395,0.009)	(-0.045,0.0114)
Occipital_Sup_L	(-0.0372,0.0576)	(-0.0001,0.00006)	(-0.0251,0.0636)	(-0.0219,0.0182)	(-0.0485,0.0123)	(-0.00002,0.00007)	(-0.0548,0.0106)	(-0.054,0.0433)
Occipital_Sup_R	(-0.0235,0.0799)	(-0.00006,0.00009)	(-0.0363,0.0496)	(-0.0247,0.0434)	(-0.0331,0.015)	(-0.00002,0.0001)	(-0.08,0.0119)	(-0.0755,0.0364)
Occipital_Mid_L	(-0.0126,0.1248)	(-0.00008,0.00012)	(-0.0299,0.0765)	(-0.0236,0.0323)	(-0.0474,0.0165)	(-0.00003,0.00015)	(-0.0521,0.0135)	(-0.0741,0.0587)
Occipital_Mid_R	(-0.0137,0.1016)	(-0.00005,0.00012)	(-0.0188,0.086)	(-0.0152,0.078)	(-0.015,0.0581)	(-0.00003,0.00021)	(-0.0511,0.0119)	(-0.0636,0.0134)
Occipital_Inf_L	(-0.0288,0.0445)	(-0.00009,0.0001)	(-0.063,0.0182)	(-0.0254,0.0336)	(-0.0207,0.013)	(-0.00002,0.00014)	(-0.0445,0.0093)	(-0.06,0.0321)
Occipital_Inf_R	(-0.015,0.0557)	(-0.00006,0.00011)	(-0.0275,0.0364)	(-0.0202,0.0463)	(-0.0176,0.0119)	(-0.00002,0.0001)	(-0.0193,0.0115)	(-0.0168,0.0115)
Fusiform_L	(-0.0857,0.0614)	(-0.00014,0.00019)	(-0.0508,0.0566)	(-0.1,0.0783)	(-0.0206,0.0764)	(-0.00004,0.00032)	(-0.0227,0.0266)	(-0.0487,0.0301)
Fusiform_R	(-0.0766,0.0649)	(-0.00009,0.00019)	(-0.0353,0.0735)	(-0.0688,0.0978)	(-0.0391,0.0249)	(-0.00003,0.00019)	(-0.0522,0.0142)	(-0.0051,0.2501)
Postcentral_L	(-0.0612,0.0234)	(-0.00018,0.00005)	(-0.0392,0.0336)	(-0.0732,0.0469)	(-0.0704,0.022)	(-0.00007,0.00004)	(-0.0391,0.0102)	(-0.1064,0.0327)
Postcentral_R	(-0.0654,0.0183)	(-0.00016,0.00005)	(-0.0428,0.0311)	(-0.0742,0.0378)	(-0.0523,0.0153)	(-0.00004,0.00008)	(-0.0537,0.0149)	(-0.0348,0.0182)
Parietal_Sup_L	(-0.0415,0.0686)	(-0.00009,0.00013)	(-0.0454,0.0446)	(-0.0243,0.0491)	(-0.0621,0.0158)	(-0.00003,0.00009)	(-0.0446,0.0124)	(-0.0822,0.0468)
Parietal_Sup_R	(-0.0458,0.0712)	(-0.00008,0.00013)	(-0.0402,0.0486)	(-0.0362,0.0548)	(-0.0199,0.0363)	(-0.00003,0.00013)	(-0.067,0.0151)	(-0.0523,0.013)
Parietal_Inf_L	(-0.0754,0.0558)	(-0.0001,0.00007)	(-0.0387,0.0456)	(-0.0625,0.0436)	(-0.0499,0.0181)	(-0.00002,0.00011)	(-0.0205,0.0158)	(-0.126,0.0141)
Parietal_Inf_R	(-0.0668,0.043)	(-0.00008,0.00007)	(-0.0255,0.051)	(-0.0489,0.0411)	(-0.0134,0.0682)	(-0.00002,0.00014)	(-0.0423,0.0105)	(-0.0147,0.0246)
SupraMarginal_L	(-0.0596,0.0394)	(-0.00005,0.0001)	(-0.0427,0.0376)	(-0.0583,0.041)	(-0.0362,0.0148)	(-0.00002,0.00006)	(-0.015,0.0212)	(-0.03,0.0176)
SupraMarginal_R	(-0.0445,0.0559)	(-0.00005,0.00009)	(-0.0285,0.05)	(-0.048,0.0432)	(-0.0134,0.0491)	(-0.00002,0.00009)	(-0.029,0.0109)	(-0.0125,0.0368)
Angular_L	(-0.046,0.045)	(-0.00003,0.00006)	(-0.0295,0.0271)	(-0.0232,0.0498)	(-0.0126,0.0417)	(-0.00002,0.00009)	(-0.0106,0.0145)	(-0.069,0.01)
Angular_R	(-0.0539,0.0437)	(-0.00004,0.00008)	(-0.0371,0.0333)	(-0.0281,0.0508)	(-0.0165,0.073)	(-0.00002,0.00011)	(-0.0285,0.0106)	(-0.0113,0.0288)
Precuneus_L	(-0.015,0.1291)	(-0.00003,0.00014)	(-0.0256,0.0657)	(-0.0222,0.0681)	(-0.0304,0.0281)	(-0.00002,0.00007)	(-0.019,0.0206)	(-0.1203,0.0291)
Precuneus_R	(-0.0387,0.0844)	(-0.00003,0.00012)	(-0.032,0.0472)	(-0.0341,0.0656)	(-0.0166,0.0514)	(-0.00002,0.00008)	(-0.0152,0.0199)	(-0.1042,0.0191)
Paracentral_Lobule_L	(-0.0509,0.0211)	(-0.00013,0.0001)	(-0.0451,0.0278)	(-0.0632,0.0465)	(-0.0508,0.0124)	(-0.00008,0.00003)	(-0.0512,0.0147)	(-0.0932,0.0223)
Paracentral_Lobule_R	(-0.0474,0.0169)	(-0.00012,0.00007)	(-0.0423,0.0216)	(-0.0556,0.0333)	(-0.0414,0.0103)	(-0.00007,0.00003)	(-0.0491,0.0139)	(-0.0412,0.0125)

Caudate_L	(-0.0288,0.0788)	(-0.00025,0.0003)	(-0.081,0.0206)	(-0.0431,0.0941)	(-0.0876,0.0107)	(-0.00006,0.00056)	(-0.012,0.0471)	(-0.0244,0.1049)
Caudate_R	(-0.0464,0.0608)	(-0.00038,0.00021)	(-0.0633,0.0325)	(-0.0427,0.1224)	(-0.0723,0.0121)	(-0.00009,0.00029)	(-0.0128,0.055)	(-0.0714,0.0233)
Putamen_L	(-0.0396,0.066)	(-0.00018,0.00033)	(-0.0562,0.0317)	(-0.0757,0.1122)	(-0.0508,0.0145)	(-0.00006,0.00039)	(-0.0188,0.0182)	(-0.03,0.1455)
Putamen_R	(-0.0643,0.0461)	(-0.00015,0.00027)	(-0.0403,0.0451)	(-0.0738,0.14)	(-0.0267,0.0224)	(-0.00003,0.00049)	(-0.0134,0.0261)	(-0.0515,0.0438)
Pallidum_L	(-0.0342,0.087)	(-0.00028,0.0005)	(-0.114,0.0305)	(-0.0649,0.0933)	(-0.071,0.0148)	(-0.00008,0.00069)	(-0.0184,0.0504)	(-0.0221,0.1254)
Pallidum_R	(-0.0593,0.0548)	(-0.00027,0.00033)	(-0.073,0.0474)	(-0.0458,0.1029)	(-0.0187,0.0334)	(-0.00006,0.0007)	(-0.0193,0.035)	(-0.0197,0.0818)
Thalamus_L	(-0.0507,0.038)	(-0.00041,0.00034)	(-0.0847,0.0375)	(-0.0921,0.0648)	(-0.0855,0.0105)	(-0.00016,0.00019)	(-0.0171,0.0905)	(-0.1479,0.0276)
Thalamus_R	(-0.0363,0.0464)	(-0.00032,0.00027)	(-0.0764,0.023)	(-0.071,0.0894)	(-0.0964,0.0109)	(-0.00031,0.00009)	(-0.0126,0.064)	(-0.1732,0.0416)
Heschl_L	(-0.0343,0.0419)	(-0.00006,0.00008)	(-0.0164,0.0383)	(-0.0438,0.0185)	(-0.0844,0.014)	(-0.00004,0.00003)	(-0.0167,0.008)	(-0.0253,0.0084)
Heschl_R	(-0.0142,0.067)	(-0.00003,0.00013)	(-0.0153,0.0398)	(-0.0136,0.0405)	(-0.0656,0.0169)	(-0.00003,0.00003)	(-0.0129,0.0095)	(-0.0171,0.0086)
Temporal_Sup_L	(-0.0483,0.066)	(-0.00007,0.00013)	(-0.0182,0.061)	(-0.0526,0.0358)	(-0.0804,0.0187)	(-0.00003,0.00008)	(-0.0203,0.0118)	(-0.0154,0.0253)
Temporal_Sup_R	(-0.0491,0.0619)	(-0.00004,0.0002)	(-0.0091,0.0947)	(-0.0581,0.042)	(-0.0307,0.0198)	(-0.00003,0.00015)	(-0.0387,0.0114)	(-0.0151,0.0638)
Temporal_Pole_Sup_L	(-0.0416,0.0932)	(-0.00008,0.00024)	(-0.0467,0.0571)	(-0.0349,0.0335)	(-0.0174,0.0715)	(-0.00004,0.00018)	(-0.0339,0.0158)	(-0.0101,0.04)
Temporal_Pole_Sup_R	(-0.0507,0.0851)	(-0.00006,0.00024)	(-0.0181,0.0923)	(-0.0459,0.0292)	(-0.0186,0.0668)	(-0.00005,0.00026)	(-0.0676,0.0126)	(-0.0162,0.0634)
Temporal_Mid_L	(-0.046,0.1029)	(-0.00006,0.00017)	(-0.032,0.0735)	(-0.0932,0.0675)	(-0.028,0.1853)	(-0.00003,0.00029)	(-0.0158,0.0357)	(-0.0396,0.1945)
Temporal_Mid_R	(-0.0419,0.0994)	(-0.00004,0.00018)	(-0.0314,0.0867)	(-0.0354,0.0931)	(-0.0646,0.2428)	(-0.00002,0.00037)	(-0.0294,0.0216)	(-0.0992,0.2395)
Temporal_Pole_Mid_L	(-0.0817,0.0406)	(-0.00016,0.00033)	(-0.0727,0.0333)	(-0.0521,0.1244)	(-0.0205,0.1246)	(-0.00008,0.00062)	(-0.0398,0.0151)	(-0.0817,0.2979)
Temporal_Pole_Mid_R	(-0.0478,0.0782)	(-0.00007,0.00038)	(-0.0486,0.0644)	(-0.0475,0.1661)	(-0.024,0.125)	(-0.00007,0.00056)	(-0.0458,0.0172)	(-0.1053,0.3112)
Temporal_Inf_L	(-0.0763,0.0353)	(-0.0002,0.00022)	(-0.0831,0.0275)	(-0.074,0.0826)	(-0.1094,0.4036)	(-0.00003,0.00126)	(0.0111,0.2303)	(-0.0735,0.8069)
Temporal_Inf_R	(-0.0699,0.0437)	(-0.00013,0.00017)	(-0.0703,0.0449)	(-0.0578,0.0821)	(-0.2302,0.5242)	(0.00005,0.00117)	(-0.0147,0.3179)	(-0.0286,0.0933)

Abbreviations: AAL, Automatic Anatomical Labeling; ReHo, Regional Homogeneity; ALFF, Amplitude of Low Frequency Fluctuation; FCD, Functional Connectivity Density; FCS, Functional Connectivity Strength.
